# Machine Learning Models for Classifying High- and Low-Grade Gliomas: A Systematic Review and Quality of Reporting Analysis

**DOI:** 10.3389/fonc.2022.856231

**Published:** 2022-04-22

**Authors:** Ryan C. Bahar, Sara Merkaj, Gabriel I. Cassinelli Petersen, Niklas Tillmanns, Harry Subramanian, Waverly Rose Brim, Tal Zeevi, Lawrence Staib, Eve Kazarian, MingDe Lin, Khaled Bousabarah, Anita J. Huttner, Andrej Pala, Seyedmehdi Payabvash, Jana Ivanidze, Jin Cui, Ajay Malhotra, Mariam S. Aboian

**Affiliations:** ^1^ Department of Radiology and Biomedical Imaging, Yale School of Medicine, New Haven, CT, United States; ^2^ Department of Neurosurgery, University of Ulm, Ulm, Germany; ^3^ Visage Imaging, Inc., San Diego, CA, United States; ^4^ Visage Imaging, GmbH., Berlin, Germany; ^5^ Department of Pathology, Yale-New Haven Hospital, Yale School of Medicine, New Haven, CT, United States; ^6^ Department of Radiology, Weill Cornell Medicine, New York, NY, United States

**Keywords:** machine learning, deep learning, artificial intelligence, glioma, systematic review

## Abstract

**Objectives:**

To systematically review, assess the reporting quality of, and discuss improvement opportunities for studies describing machine learning (ML) models for glioma grade prediction.

**Methods:**

This study followed the Preferred Reporting Items for Systematic Reviews and Meta-Analyses of Diagnostic Test Accuracy (PRISMA-DTA) statement. A systematic search was performed in September 2020, and repeated in January 2021, on four databases: Embase, Medline, CENTRAL, and Web of Science Core Collection. Publications were screened in Covidence, and reporting quality was measured against the Transparent Reporting of a multivariable prediction model for Individual Prognosis Or Diagnosis (TRIPOD) Statement. Descriptive statistics were calculated using GraphPad Prism 9.

**Results:**

The search identified 11,727 candidate articles with 1,135 articles undergoing full text review and 85 included in analysis. 67 (79%) articles were published between 2018-2021. The mean prediction accuracy of the best performing model in each study was 0.89 ± 0.09. The most common algorithm for conventional machine learning studies was Support Vector Machine (mean accuracy: 0.90 ± 0.07) and for deep learning studies was Convolutional Neural Network (mean accuracy: 0.91 ± 0.10). Only one study used both a large training dataset (n>200) and external validation (accuracy: 0.72) for their model. The mean adherence rate to TRIPOD was 44.5% ± 11.1%, with poor reporting adherence for model performance (0%), abstracts (0%), and titles (0%).

**Conclusions:**

The application of ML to glioma grade prediction has grown substantially, with ML model studies reporting high predictive accuracies but lacking essential metrics and characteristics for assessing model performance. Several domains, including generalizability and reproducibility, warrant further attention to enable translation into clinical practice.

**Systematic Review Registration:**

PROSPERO, identifier CRD42020209938.

## 1 Introduction

Gliomas are the most common primary brain malignancy (1). They are classified according to histopathologic and molecular World Health Organization (WHO) criteria: grades 1/2 (low-grade gliomas (LGG) and grades 3/4 [high-grade gliomas (HGG)] (2). Glioblastomas, WHO grade 4 tumors, are the most aggressive with a 15-month median overall survival (3).

Because prognosis (3, 4) and treatment (5) vary with glioma grade, accurate classification is essential for guiding clinical decision-making and mitigating risks posed by unnecessary or delayed surgery due to misdiagnosis (6). The gold standard for diagnosis, histopathology, requires surgical resection or stereotactic biopsy for analysis. These invasive procedures, however, carry significant risks and complications (7). Gliomas also exhibit intratumoral heterogeneity with associated sampling error (8). Therefore, a need exists for timely pre-operative whole-glioma grading. As a non-invasive tool for analyzing entire lesions, imaging overcomes the limitations of diagnostic surgical procedures. Although conventional MRI has had modest success in glioma grading (sensitivity 55-83%) (9), the diagnostic potential of imaging has expanded with the use of advanced imaging, radiomics, and artificial intelligence.

Radiomics quantitatively characterizes medical images using image-derived features that serve as biomarkers for tumor phenotypes (10). Artificial intelligence technologies, such as machine learning (ML), have augmented radiomics. By leveraging robust high-dimensional data, ML enhances predictive performance (11). Deep learning (DL) is a subtype of ML that has sparked recent interest given its superior performance in image analysis and suitability for high volumes of data (12). For imaging applications, DL generates useful outputs from input images using multilayer neural networks. Convolutional Neural Networks are the primary DL architecture for image classification (13).

In clinical practice, ML models may increase the value of diagnostic imaging and enhance patient management, for example, by motivating earlier grade-appropriate interventions (14, 15). Despite these opportunities, ML has not been implemented clinically because of numerous technical (data requirements, need for training, low standardization and interpretability) and non-technical (ethical, financial, legal, educational) barriers (16).

High-quality scientific reporting is necessary for readers to critically interpret or replicate studies and encourage translation into practice. Prior work indicates that reporting quality in prediction studies is poor (17). To address this, the Transparent Reporting of a multivariable model for Individual Prognosis or Diagnosis (TRIPOD) Statement was published in 2015 (18). Most of TRIPOD is applicable to ML-based prediction model studies; however, ML-specific guidelines are lacking. The need for such guidelines has initiated development of a TRIPOD extension for ML-based prediction models (TRIPOD-AI) (19, 20).

While ML demonstrates promise for accurate glioma grading, few works have characterized the state of ML in glioma grade prediction (21–23). A systematic review of the literature can identify potential ML methods for clinical use and generate insights for implementation. This study aims to (1) systematically review and synthesize the body of literature using ML for classification of glioma grade, (2) evaluate study reporting quality using TRIPOD, and (3) discuss opportunities for bridging the ML bench-to-clinic implementation gap.

## 2 Materials and Methods

This study followed the guidelines in the Preferred Reporting Items for Systematic Reviews and Meta-Analyses of Diagnostic Test Accuracy (PRISMA-DTA) statement (24) and was registered with the International Prospective Register of Systematic Reviews (PROSPERO, CRD42020209938). An institutional librarian searched the literature published through September 18, 2020, using four databases: Cochrane Central Register of Controlled Trials, EMBASE, Medline, and Web of Science Core Collection. A multi-database approach was pursued because prior work has demonstrated that a single database search may omit pertinent studies (25). Keywords and controlled vocabulary included the following terms and combinations thereof: “artificial intelligence,” “machine learning,” “deep learning,” “radiomics,” “magnetic resonance imaging,” “glioma,” and related terms. The search was repeated on January 29, 2021, to gather additional articles published through this date. A full search strategy is provided in **Appendix A1 (Supplementary)**. A second institutional librarian reviewed the search prior to execution.

**Figure 1:**
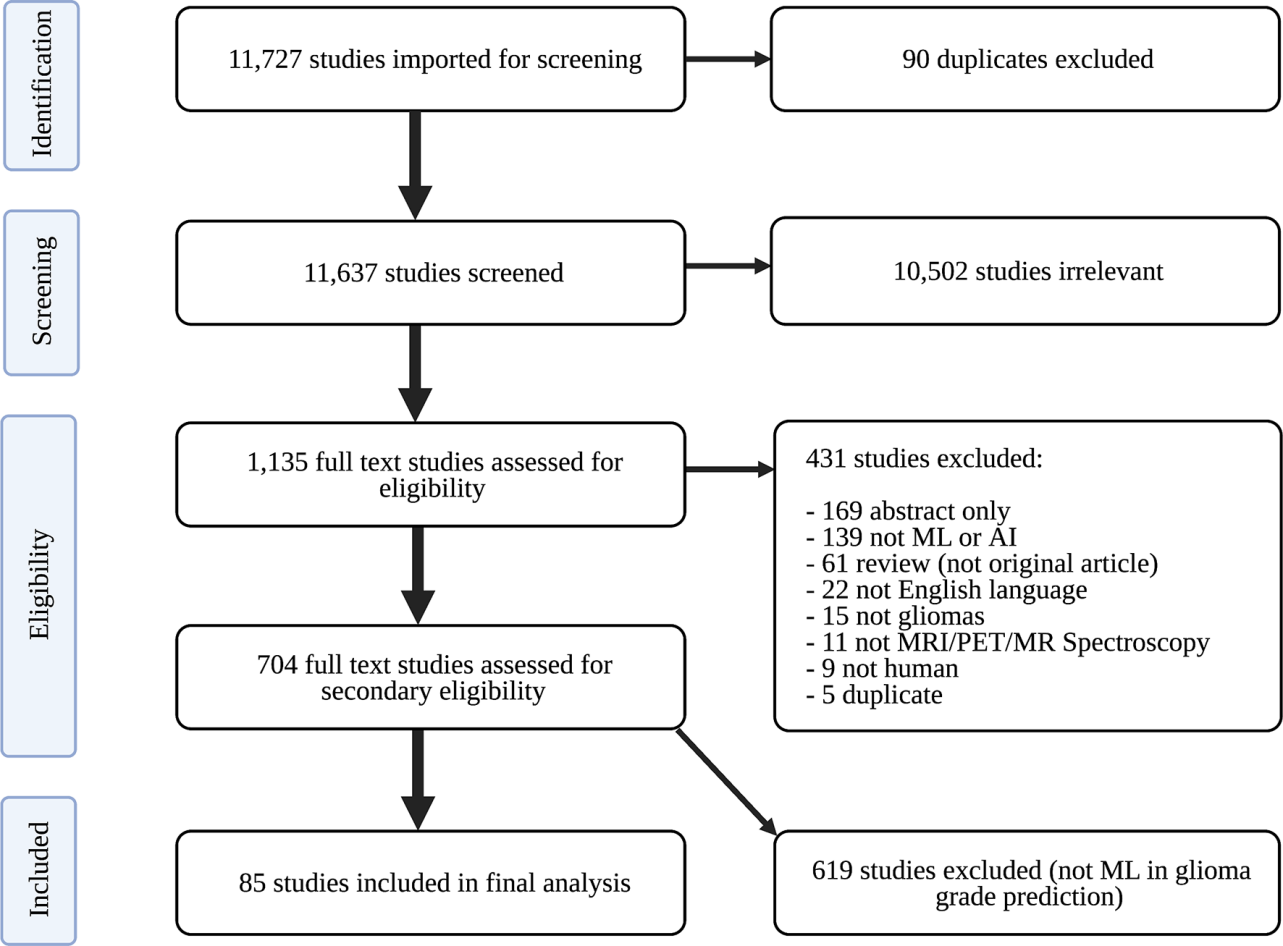
PRISMA flow diagram of study search strategy. ML, machine learning; PRISMA, Preferred Reporting Guidelines for Systematic Reviews and Meta-Analyses.

Only peer-reviewed studies were imported into Covidence (Veritas Health Innovation Ltd) for screening. Covidence is an online tool designed to streamline the systematic review process. Duplicate studies were identified and removed. Study abstracts were then screened for relevance to neuro-oncology by two of three independent reviewers: an experienced board-certified neuroradiologist, radiology resident, and graduate student in artificial intelligence. The board-certified neuroradiologist resolved discrepancies in screening recommendations. Relevant articles were subsequently assessed for eligibility. To ensure completeness, appropriateness, and understandability of eligible studies, the following exclusion criteria were established: (1) abstract-only; (2) not primary literature; (3) non-English; (4) unrelated to artificial intelligence; (5) unrelated to gliomas; (6) unrelated to imaging; (7) non-human research subjects; and (8) duplicates. Studies were not excluded based on publication year in order to have comprehensive analysis of historical and contemporary literature. Eligible studies underwent full text review to identify those using ML to classify gliomas by grade. Studies exclusively developing predictive models with distinct focuses (e.g., predicting glioma IDH status, glioma segmentation) were not included in analysis. A PRISMA flow diagram describing our study selection process is depicted in **Figure 1**.

Whole data was independently extracted by two trained medical student researchers using a standardized Microsoft Excel (Microsoft Corporation) form. Conflicts were resolved through team discussion and consensus. When necessary, articles were carefully re-reviewed to obtain missing information after data extraction. The following data points were extracted: article characteristics (title, lead author, country of lead author, publication year), data characteristics (data source, country (or countries) of data acquisition, dataset size, types and number of tumors for training/testing/validation, model validation technique), grading characteristics (study definition of HGG and LGG, gold standard for glioma grading), model characteristics (best performing ML classifier, classification task, supervised/semi-supervised/unsupervised learning, types of features in classifier, imaging sequences used by classifier, measures of classifier performance) and reporting characteristics (TRIPOD items, explained below).

Reporting quality was assessed against the TRIPOD statement in agreement with the TRIPOD adherence assessment form (26) and author explanations (18, 27). TRIPOD contains 20 main items (e.g., main item 5) that apply to studies developing prediction models, 10 of which contain subitems (e.g., 5a, 5b, 5c). Among the 30 total items that can be evaluated and scored, three (item 5c, 11, 14b) were excluded because they were not applicable to our studies. The remaining 27 items were scored for every study. Each item includes one or more elements, all of which must score a “yes” for the item to score “1.” To calculate a study’s adherence rate to TRIPOD, the number of items scoring “1” was divided by the total number of scored items for the study. Adherence rate for a given TRIPOD item across all studies was calculated by dividing the number of studies scoring “1” for that item by the total number of studies scored.

TRIPOD adherence rates and descriptive statistics (e.g., frequencies, mean ± standard deviation) were calculated and displayed with GraphPad Prism 9 (GraphPad Software). GraphPad Prism 9 is a scientific graphing and statistical software supporting data analysis. Descriptive statistics were obtained to summarize study characteristics, dataset and model characteristics, features, imaging modalities, and model prediction performance, among other domains. Only the best performing classifiers’ performance metrics (accuracy, AUC, sensitivity, specificity, positive predictive value, negative predictive value, and F1 score) defined in each study are presented. Best performing classifiers were determined based on accuracy results. In the few instances when accuracy was not reported, AUC determined the best performing classifier. All studies meeting our inclusion criteria ((1) identified by search strategy; (2) relevant to neuro-oncology; (3) not excluded during eligibility assessment; and (4) clearly evaluate ML-based classification of glioma grade) contributed to the sample size of our study. References for included studies are listed in **Appendix A4 (Supplementary)**.

## 3 Results

### 3.1 Study Characteristics

The search identified 11,727 candidate articles, with 11,637 studies screened for relevance to neuro-oncology. Agreement between screeners was substantial [(Cohen’s kappa: 0.77 ± 0.04, see **Table A1 (Supplementary)**]. 1,135 articles underwent full text review, and 85 articles were included in analysis (**Figure 1**).

**Figure 2:**
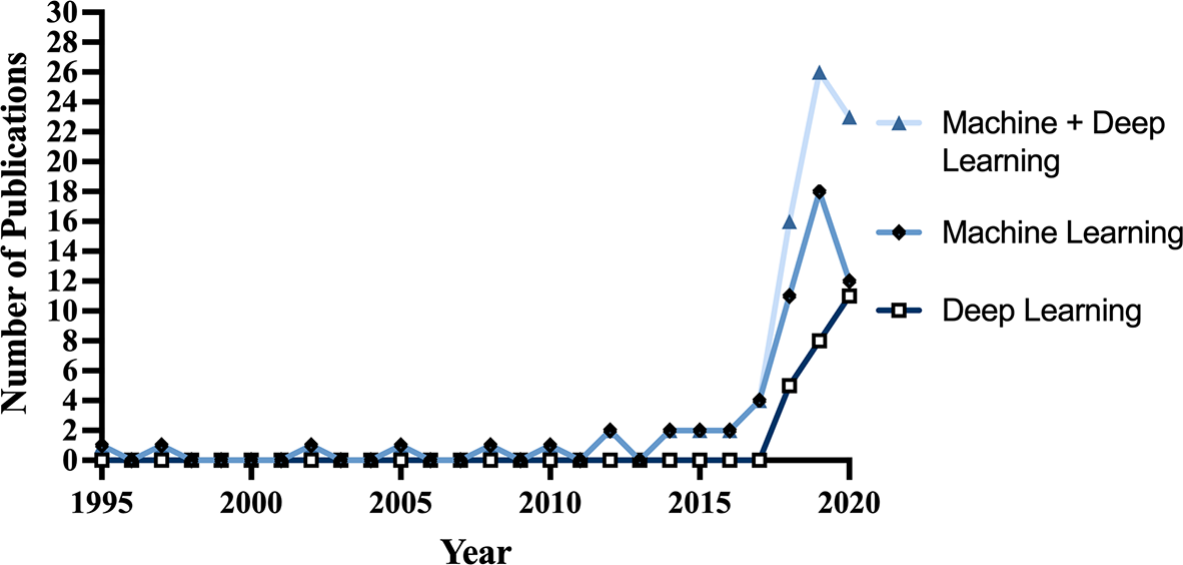
Number of studies published per year from 1995-2020.

**Figure 3:**
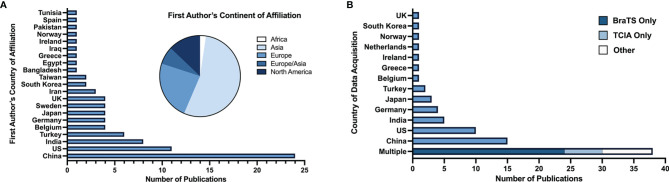
**(A)** Number of studies by first author’s country of affiliation and respective continent. **(B)** Number of studies by country (or countries) of data acquisition.

67 articles (79%) were published between 2018 and 2021, with 26 articles (31%) published in 2019 alone (**Figure 2**). Based on lead author affiliations, most articles were from China, the US, or India (n=45, 51%) (**Figure 3**).

**Figure 4:**
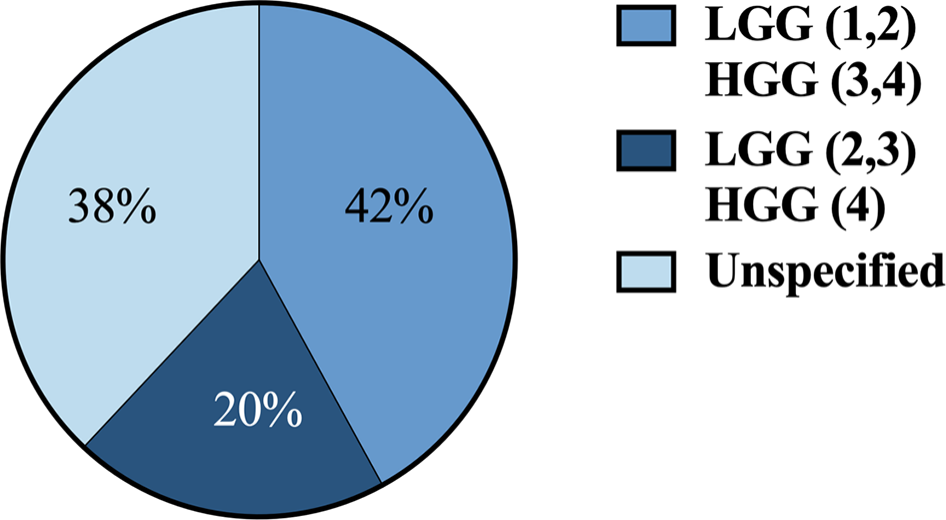
Classification systems used across studies for defining HGG vs. LGG by grade 1-4. HGG, high-grade gliomas; LGG, low-grade gliomas.

36 articles (42%) defined HGG as grade 3 and 4 and LGG as grade 1 and 2. 17 articles (20%) defined HGG as grade 4 and LGG as grades 2 and 3. 32 articles (38%) didn’t define grades for HGG and LGG (**Figure 4**).

### 3.2 Study Findings

#### 3.2.1 Dataset and Model Characteristics

Among the 84 articles with identifiable patient data sources, data was most commonly acquired multi-nationally (n=38, 45%), entirely in China (n=15, 18%), or entirely in the US (n=10, 12%) (**Figure 3**). BraTS (28) and TCIA (29) datasets, which are publicly available multi-institutional datasets containing multi-parametric MRI scans, were used in 45% (n=38) of studies. Conventional ML was the primary ML model for glioma grade prediction in 59 (69%) studies and DL in 26 (31%) studies. Of all 85 studies included, 80 (94%) reported the number of patients in their datasets (mean: 177 ± 140). Studies developing conventional ML models reported mean dataset sizes of 168 ± 150 patients. DL studies reported mean dataset sizes of 199 ± 109 patients. Among the 67 studies whose best performing models were binary classifiers of HGG and LGG and reported the number of HGG and LGG used in model development, 58 (87%) had imbalanced datasets characterized by an unequal number of HGG and LGG patients. Most studies (n=44, 66%) used datasets containing more HGG than LGG patients (i.e., HGG : LGG ratio >1). 14 studies (21%) had fewer HGG than LGG patients (HGG : LGG ratio <1).

Only 5 (6%) studies reported external validation. Of the 80 other studies, 68 (85%) reported internal validation and 12 (15%) did not clearly report validation methods. 82 (96%) of studies had supervised learning algorithms and 3 (4%) used semi-supervised learning. No studies reported unsupervised learning algorithms. The gold standard for glioma grading was histopathology in all studies.

#### 3.2.2 Features

Texture (second-order) features and first-order features were the most common feature subsets, extracted in 45 (53%) and 42 (49%) studies, respectively. Shape and/or size features (n=28, 33%) and DL extracted features (n=20, 24%) were also common. Hemodynamic (n=5), qualitative (n=6), higher-order (n=4) and spectroscopic features (n=8) were observed in less than 10% of studies. Definitions for feature types are provided in **Table A7 (Supplementary)**.

#### 3.2.3 Imaging Modalities

T1-weighted contrast-enhanced (T1CE) imaging was the most common sequence used in best performing models (n=54, 64%), followed by T2 (n=46, 54%) and FLAIR (n=40, 47%). T1 pre-contrast was less common (n=35, 41%). Perfusion-weighted imaging (n=15), MR Spectroscopy (n=9) and diffusion-weighted imaging (n=12) were used in 11-18% of models. PET and fMRI were only used in one model each.

#### 3.2.4 Prediction Performance

A summary of model performance measures across studies is shown in **Table 1**. The mean glioma grade prediction accuracy of the best performing algorithm per study was 0.89 ± 0.09. This parameter was determined by taking the prediction accuracy of the best performing algorithm in each study for all studies and calculating a mean value and standard deviation. Lower accuracies were reported for models undergoing external validation (mean: 0.82 ± 0.09, n=5). DL models had a mean prediction accuracy of 0.92 ± 0.08 and conventional ML models 0.88 ± 0.09.

**Table 1:** Mean (± standard deviation) aggregate performance metrics across studies.

Accuracy (n=82)	AUC (n=48)	Sensitivity (n=55)	Specificity (n=51)	Positive Predictive Value (n=12)	Negative Predictive Value (n=6)	F1 Score (n=7)
0.89 ± 0.09	0.92 ± 0.07	0.89 ± 0.09	0.88 ± 0.11	0.90 ± 0.09	0.82 ± 0.08	0.89 ± 0.11
(0.53-1.00)	(0.73-1.00)	(0.63-1.00)	(0.55-1.00)	(0.68-1.00)	(0.73-0.94)	(0.67-0.98)

n, number of studies reporting metric.

**Figure 5:**
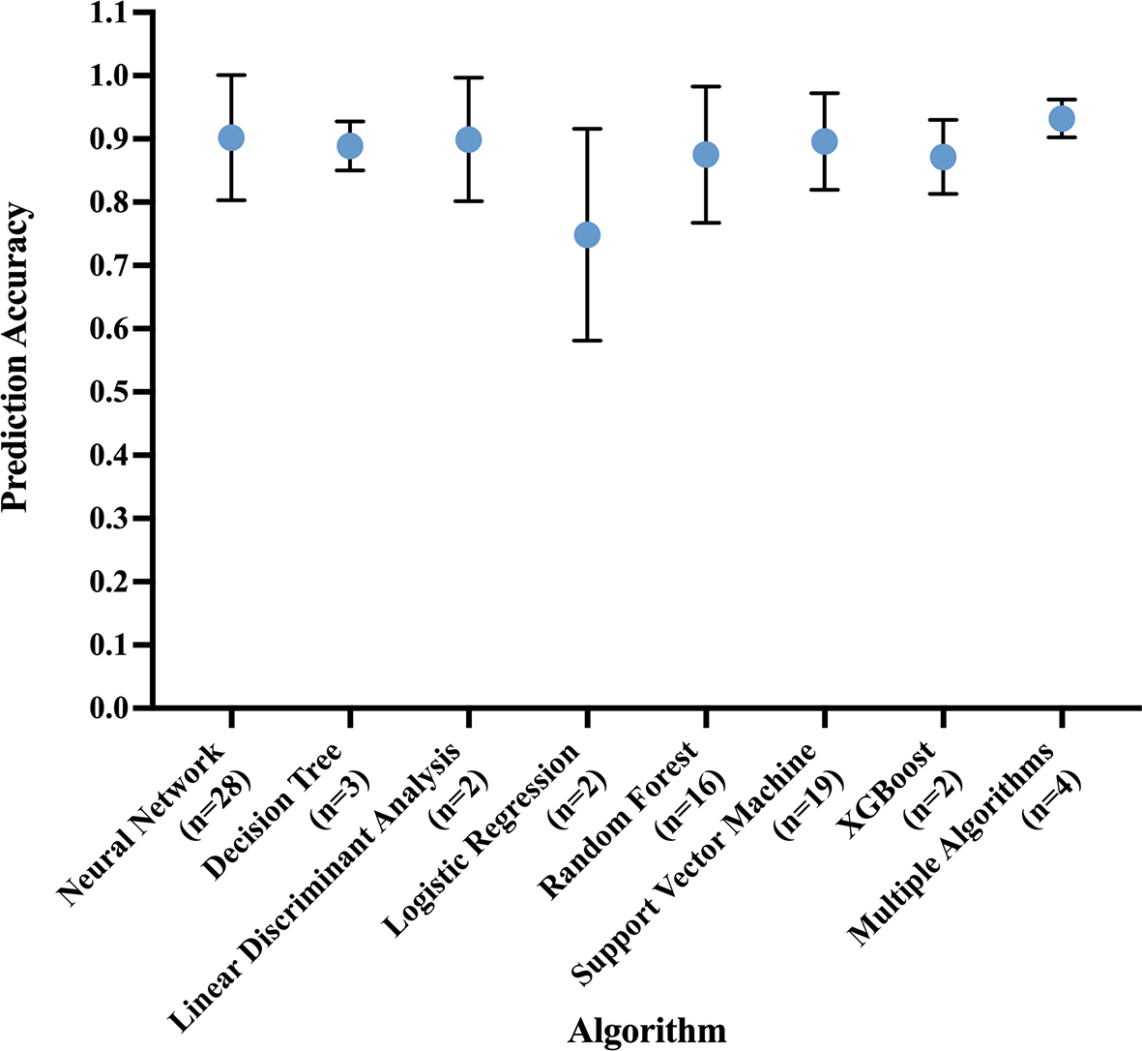
Prediction accuracy of most common algorithm types, measured in the best performing algorithm of each study. Circle at mean. Error bars indicate standard deviation.

The most common best performing conventional ML model was Support Vector Machine (mean accuracy: 0.90 ± 0.07) and DL model was Convolutional Neural Network (mean accuracy: 0.91 ± 0.10) (**Figure 5**).

We grouped all studies by data source into 4 categories: BraTS, TCIA, single center, and multicenter (excluding BraTS and TCIA) data. Studies which used BraTS as a data source had a mean accuracy of 0.93 ± 0.04 (n=27) and studies using TCIA had a mean accuracy of 0.91 ± 0.08 (n=12). Single center datasets were the most common (n=43) with a mean accuracy of 0.88 ± 0.07, and multicenter hospital datasets the least common (n=6, mean accuracy: 0.80 ± 0.18).

We additionally identified studies whose models were built on relatively large (n≥200) datasets and externally validated, two characteristics indicating potential generalizability. Only one study (1%) had both characteristics (accuracy: 0.72) (30). Further analysis of model performance by dataset source, dataset size, validation technique, and glioma grade classification task can be found in **Appendix A2** and **Tables A2-A5 (Supplementary)**. Characteristics of the 10 studies reporting the highest accuracy results for their best performing algorithms are summarized in **Table 2**. Characteristics of all included studies may be seen in **Table A6 (Supplementary)**.

**Table 2:** Characteristics of the 10 studies reporting the highest accuracy results for their best performing models, including: glioma grade classification task, dataset source and size, ratio of high- to low-grade gliomas, validation technique, imaging sequences used in prediction, feature types used in prediction, best performing algorithm (based on accuracy results), and performance metrics.

Paper	Glioma Grade Classification Task	Dataset	HGG : LGG Ratio	Validation Technique	Imaging Sequences	Features	Best Algorithm	Performance Metrics
Hedyehzadeh et al. (2020) (31)	2/3 vs. 4	TCIA (n=461 patients)	1.3:1 (262 HGG, 199 LGG in total set)	Internal (4-fold cross-validation)	T1, T1CE, T2, FLAIR	Texture	Support Vector Machine	Accuracy = 1.00 Sensitivity = 1.00 Specificity = 1.00
BashirGonbadi and Khotanlou (2019) (32)	1/2 vs. 3/4	BraTS (n=285 patients)	2.8:1 (210 HGG, 75 LGG in total set)	Internal (Holdout, 15% of dataset)	T1, T1CE, T2, FLAIR	Deep learning extracted	Convolutional Neural Network	Accuracy = 0.9918
Polly et al. (2018) (33)	HGG vs. LGG (unclear)	BraTS (n=160 images)	1:1 (50 HGG, 50 LGG in testing set)	Unspecified	T2	First-order, Shape, Texture	Support Vector Machine	Accuracy = 0.99 Sensitivity = 1.00 Specificity = 0.9803
De Looze et al. (2018) (34)	HGG vs. LGG (unclear)	Single center hospital (n=381 patients)	Unclear	Internal (5-fold cross-validation)	T1, T1CE, T2, FLAIR, Diffusion	Qualitative	Random Forest	Accuracy = 0.99 AUC = 0.99 Sensitivity = 1.00 Specificity = 0.92
Sharif et al. (2020) (35)	HGG vs. LGG (unclear)	BraTS (n=30 patients)	2.3:1 (7 HGG, 3 LGG in testing set)	Internal (Holdout, 10-fold cross-validation)	T1, T1CE, T2, FLAIR	Deep learning extracted	Convolutional Neural Network	Accuracy = 0.987
Muneer et al. (2019) (36)	1 vs. 2 vs. 3 vs. 4	Single center hospital (n=20 patients)	1.3:1.6:1:1.5 (39 grade 1, 51 grade 2, 31 grade 3, 47 grade 4 images in testing set)	Internal (Holdout, 30% of dataset)	T2	Deep learning extracted	VGG19 (Deep Convolutional Neural Network)	Accuracy = 0.9825 Sensitivity = 0.9272 Specificity = 0.9813 Positive Predictive Value = 0.9471 F1 Score = 0.9371
Dandil and Bicer (2020) (37)	1/2 vs. 3 vs. 4 vs. meningioma	INTERPRET (n=179 patients)	Unclear	Unspecified	MR Spectroscopy (Time of Echo 20ms and 136ms)	First-order, Shape and size, Texture	Long Short-Term Memory (Neural Network)	Accuracy = 0.982 AUC = 0.9936 Sensitivity = 1.00 Specificity = 0.9753
Tian et al. (2018) (38)	2 vs. 3/4	Single center hospital (n=153 patients)	2.6:1 (111 HGG, 42 LGG in total set)	Internal (10-fold cross-validation)	T1, T1CE, T2, Diffusion, Perfusion (3D Arterial Spin Labeling)	Texture	Support Vector Machine	Accuracy = 0.981 AUC = 0.992 Sensitivity = 0.987 Specificity = 0.974
Lo et al. (2019) (39)	2 vs. 3 vs. 4	TCIA (n=130 patients)	1:1.4:1.9(30 grade 2, 43 grade 3 and 57 grade 4 in total set)	Internal (10-fold cross-validation)	T1CE	Deep learning extracted	Deep Convolutional Neural Network	Accuracy = 0.979 AUC = 0.9991
Kumar et al. (2020) (40)	1/2 vs. 3/4	BraTS (n=285 patients)	2.8:1 (210 HGG, 75 LGG in total set)	Internal (5-fold cross-validation)	T1, T1CE, T2, (T2W)-FLAIR	First-order, Shape, Texture	Random Forest	Accuracy = 0.9754 AUC = 0.9748 Sensitivity = 0.9762 Specificity = 0.9733 F1 Score = 0.983

Testing or validation metrics are reported when available, otherwise training metrics are reported. HGG, high-grade gliomas; LGG, low-grade gliomas; ML, machine learning; PRISMA-DTA, Preferred Reporting Items for Systematic Reviews and Meta-Analyses of Diagnostic Test Accuracy; T1CE, T1-weighted contrast-enhanced; TRIPOD, Transparent Reporting of a multivariable prediction model for Individual Prognosis Or Diagnosis.

### 3.3 Quality Assessment

**Figure 6:**
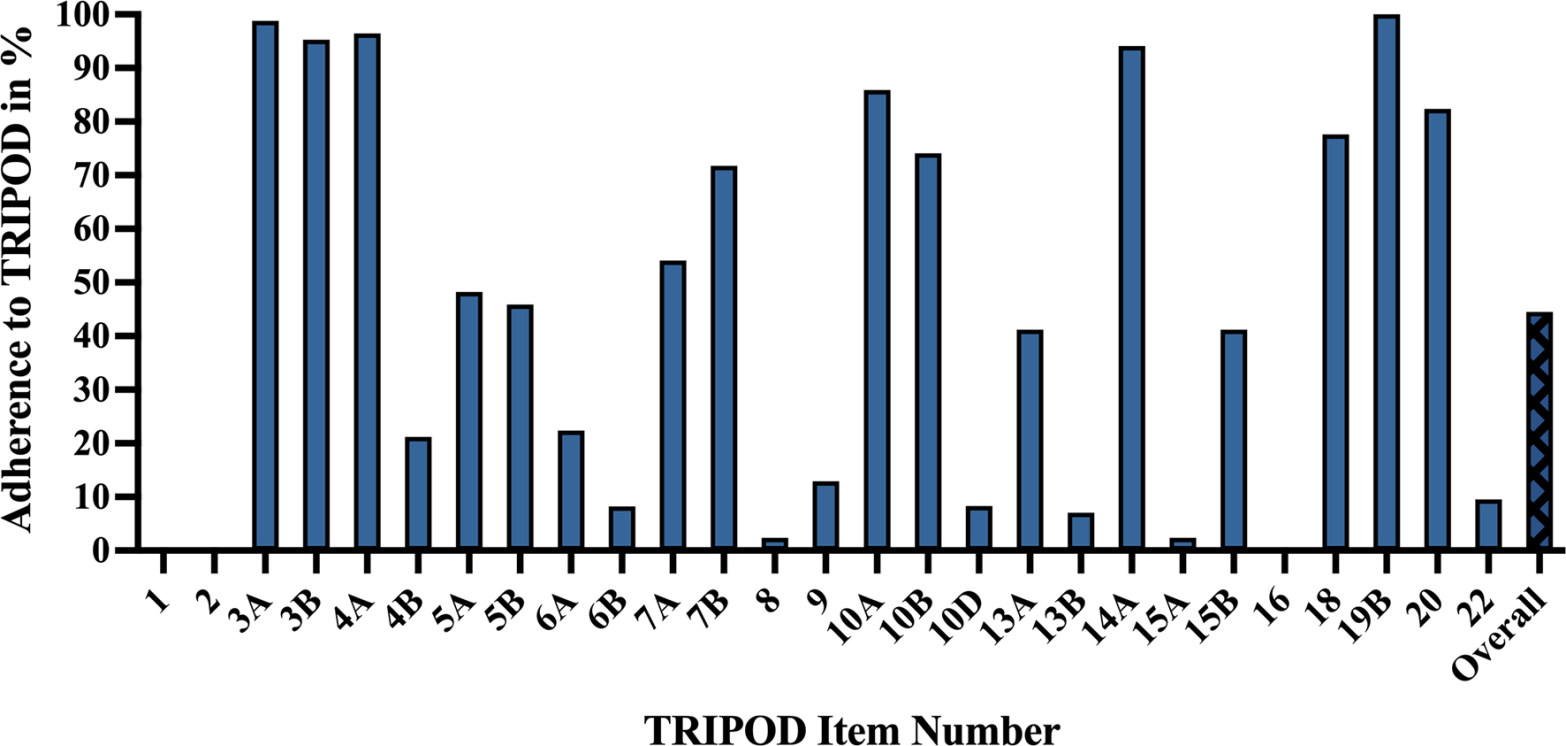
TRIPOD adherence of machine learning glioma grade prediction studies. Adherence rate for individual items represents the percent of studies scoring a point for that item: 1 – title. 2 – abstract. 3a – background. 3b – objectives. 4a – study design. 4b – study dates. 5a – study setting. 5b – eligibility criteria. 6a – outcome assessment. 6b – blinding assessment of outcome. 7a – predictor assessment. 7b – blinding assessment of predictors. 8 – sample size justification. 9 – missing data. 10a – predictor handling. 10b – model type, model-building, and internal validation. 10d – model performance. 13a – participant flow and outcomes. 13b – participant demographics and missing data. 14a – model development (participants and outcomes). 15a – full model specification. 15b – using the model. 16 – model performance. 18 – study limitations. 19b – results interpretation. 20 – clinical use and research implications. 22 – funding. Overall – mean TRIPOD adherence rate of all studies. TRIPOD, Transparent Reporting of a multivariable prediction model for Individual Prognosis Or Diagnosis.

The mean adherence rate to TRIPOD was 44.5% ± 11.1%, with poor reporting adherence in categories including model performance (0%), abstract (0%), title (0%), justification of sample size (2.4%), full model specification (2.4%), and participant demographics and missing data (7.1%). High reporting adherence was observed for results interpretation (100%), background (98.8%), study design/source of data (96.5%), and objectives (95.3%) (**Figure 6**).

Titles (0%) did not identify the development of prediction models. Abstracts (0%) frequently lacked source of data, overall sample size, and calibration methods. Regarding model performance (0%), very few studies reported measures for model calibration or confidence intervals. Most studies failed to specify model regression coefficients (2.4%) or provide justifications of sample size (2.4%), e.g., how sample size was arrived at according to statistical or practical grounds. More detailed explanations of TRIPOD items and their adherence rates can be found in **Table A8 (Supplementary)**.

## 4 Discussion

Our systematic review analyzed 85 articles describing ML applications for glioma grade prediction and revealed several trends. First, the number of studies published per year grew steadily between 2016 and 2019. Second, imaging sequences and ML models became less conventional, with the emergence of advanced MRI sequences (MR Spectroscopy, Perfusion) in the early 2000s (41) and DL models in 2018 (42). Third, datasets recently expanded to encompass multiple institutions, with BraTS and TCIA datasets appearing in 2017. While ML model studies report high predictive accuracies, they underreport critical model performance measures, lack a common validation dataset, and vary remarkably in glioma classification systems, ML algorithms, feature types and imaging sequences used for prediction, etc., limiting model comparison. Here, we identify several opportunities for improvement to prepare models for multicenter clinical adoption.

### 4.1 Study Datasets, Validation Techniques, Classification Systems, and Reporting Quality

Prior to broad clinical use, ML models must be trained and validated on large, multi-institutional datasets to ensure generalizability (43). Dataset sizes, however, were low in our study, and most publications lacked external validation. These findings are consistent with those from a similar systematic review by Tabatabaei et al. (23). Moreover, while studies based on highly curated datasets, including BraTS or TCIA, showed consistently high accuracy results, algorithms trained on these datasets without external validation may not have reproducible results in clinical practice, where imaging protocols are less standardized, image quality is variable, and tumor presentations are heterogeneous. To show that models perform well across distinct populations and are fit for broad clinical implementation, future works should use sizable, less-curated, multicenter datasets and externally validate their models.

ML models should also be trained according to standardized definitions of glioma grade. Interestingly, definitions were variable for HGG and LGG, with some studies considering grade 3 gliomas to be high-grade and others low-grade. Lack of a unified classification system may hinder predictive model performances on external datasets, given that the images used for segmentation, feature extraction, and model training/testing are labeled HGG or LGG based on non-uniform definitions. As glioma grade guides clinical management, it is essential that algorithm outputs of “HGG” and “LGG” reflect a universal definition consistent with current WHO criteria.

An alternative to binary high-or-low grading is to report numerical glioma grades (1, 2, 3, or 4) and tumor entities. Importantly, grade and entity classifications are evolving. In 2016, purely histopathological classification was succeeded by classification based on both molecular and histopathologic parameters (44). In 2021, cIMPACT-NOW established further changes to glioma grading, for example, by redefining GBM to be an IDH (Isocitrate Dehydrogenase)-wild-type lesion distinct from IDH-mutant grade 4 astrocytomas (45, 46). Classification changes may have led to inconsistencies in tumor entities and grades reported in glioma grade prediction studies across the years, limiting model comparison. As WHO criteria continue to evolve and affect generalizability of study results, we recommend that studies clearly reference the criteria used in glioma grading, report the glioma entities and corresponding grade used in model development, and describe the predictive performances by both entity and grade. This will promote comprehensible and traceable results over time. Moreover, integrated techniques that characterize disease according to both radiological and biological features are emerging in neuro-oncology (47). We advise future researchers to consider the implementation of these techniques into ML model development studies predicting glioma grade, molecular markers, response to treatment, prognosis, and other applications within neuro-oncology.

Finally, reporting of ML models should be transparent, thorough, and reproducible to facilitate proper assessment for use in clinical practice (48). Several comprehensive checklists are used to assess reporting quality of diagnostic models, including Checklist for Artificial Intelligence in Medical Imaging (49) and TRIPOD. In our study, mean adherence to TRIPOD was low, with key assessment elements such as model performance scoring poorly. These findings reflect inadequate study reporting. To address this, we recommend future studies use appropriate reporting frameworks to guide all phases of study execution, from initial design through manuscript writing. For ML studies, the relevance of TRIPOD as a benchmark for reporting quality may be questioned. Published explanations and elaborations of TRIPOD focus on regression-based models, a shortcoming that TRIPOD authors have recently acknowledged (19). We support their initiative to create a TRIPOD Statement specific to ML (TRIPOD-AI) (19, 20), and in the context of this work, to improve the reporting quality of literature concerning ML in glioma grade prediction.

### 4.2 Limitations

This study has several limitations. First, the timing and criteria of our search may have missed relevant studies (e.g., recent and unpublished works). Moreover, 191 of the 1,135 (16.8%) studies assessed for eligibility were excluded because they were abstracts (n=169, 14.9%) or not in English (n=22, 1.9%), creating a potential selection bias. However, full texts were required for complete data extraction and quality of reporting analysis, and we unfortunately did not have the resources to translate non-English articles. Second, we determined best performing algorithms based on accuracy, which excluded the three studies that did not report accuracy results for their models. Accuracy, furthermore, may be considered a flawed performance metric for ML models applied to imbalanced datasets (50), which constituted most datasets in our study. With imbalanced datasets, ML models intrinsically overfit toward the majority class, risking higher misclassification rates for minority classes (51, 52). Because accuracy may be high even if a minority class is poorly predicted, we recommend study authors consistently report a full slate of model performance metrics. Including metrics sensitive to performance differences within imbalanced datasets (e.g., AUC) (53) will enable a more thorough assessment of ML model performance. Third, the inconsistent definitions for HGG and LGG, evolving grading criteria, high heterogeneity of our included articles and low number of articles reporting confidence intervals for their performance metrics limited the pooling of results across studies and subsequent generation of conclusions. As a result, we could not perform a meta-analysis (54, 55).

## 5 Conclusion

The application of ML to glioma grade prediction has grown substantially, with ML model studies reporting high predictive accuracies but lacking essential metrics and characteristics for assessing model performance. To increase the generalizability, standardization, reproducibility, and reporting quality necessary for clinical translation, future studies need to (1) train and test on large, multi-institutional datasets, (2) validate on external datasets, (3) clearly report glioma entities, corresponding glioma grades, and a full state of predictive performance metrics by both grade and entity, and (4) adhere to reporting guidelines.

## Data Availability Statement

The original contributions presented in the study are included in the article/**Supplementary Material**, further inquiries can be directed to the corresponding author.

## Author Contributions

All authors listed have made a substantial, direct, and intellectual contribution to the work and approved it for publication.

## Funding

SM receives funding in part from the Biomedical Education Program (BMEP). RB receives funding in part from the National Institute of Diabetes and Digestive and Kidney Disease of the National Institutes of Health under Award Number T35DK104689. MA received funding from American Society of Neuroradiology Fellow Award 2018. This publication was made possible by KL2 TR001862 (MA) from the National Center for Advancing Translational Science (NCATS), components of the National Institutes of Health (NIH), and NIH roadmap for Medical Research. Seyedmehdi Payabvash has grant support from NIH/NINDS K23NS118056, foundation of American Society of Neuroradiology (ASNR) #1861150721, Doris Duke Charitable Foundation (DDCF) #2020097, and NVIDIA. Funders were not involved in the study design, collection, analysis, interpretation of data, the writing of this article or the decision to submit it for publication.

## Author Disclaimer

The content is solely the responsibility of the authors and does not necessarily represent the official views of the National Institutes of Health.

## Conflict of Interest

Author ML is an employee and stockholder of Visage Imaging, Inc., and unrelated to this work, receives funding from NIH/NCI R01 CA206180 and is a board member of Tau Beta Pi engineering honor society. KB is an employee of Visage Imaging, GmbH. JI has funding support for an investigator-initiated clinical trial from Novartis Pharmaceuticals (unrelated to this work).

The remaining authors declare that the research was conducted in the absence of any commercial or financial relationships that could be construed as a potential conflict of interest.

## Publisher’s Note

All claims expressed in this article are solely those of the authors and do not necessarily represent those of their affiliated organizations, or those of the publisher, the editors and the reviewers. Any product that may be evaluated in this article, or claim that may be made by its manufacturer, is not guaranteed or endorsed by the publisher.
