# Supplementary material for: Machine Learning Models for Classifying High- and Low-Grade Gliomas: A Systematic Review and Quality of Reporting Analysis
Source: Front Oncol. 2022 Apr 22;12:856231. doi: 10.3389/fonc.2022.856231 (PMC9076130; doi:10.3389/fonc.2022.856231)
Supplement: Supplementary file 1 [file Table_1.docx]

Supplementary Material

**Appendix A1. Study Search Strategy.**

**Embase** <1974 to 2021 January 29>

1 exp Artificial Intelligence/ 45007

2 machine learning/ 37007

3 deep learning/ 12393

4 ((artificial* or machine* or deep*) adj3 (intelligence or learning)).tw,kw. 73708

5 AI.ti,ab. 39008

6 exp computer assisted diagnosis/ 1169775

7 computer* assist* diagnosis.tw,kw. 937

8 radiomics/ 1903

9 radiomic*.tw,kw. 4867

10 or/1-9 1305625

11 exp nuclear magnetic resonance imaging/ 1001848

12 (Magnetic Resonance Imag* or MR-Imag* or MR Imag or MRI* or NMR).tw,kw. 882389

13 11 or 12 1278307

14 exp glioma/ 139715

15 glioma*.tw,kw. 84577

16 (glial adj2 (tumor* or tumour*)).tw,kw. 3616

17 (glioblastoma* or astrocytoma* or astrocytic glioma* or astroglioma).tw,kw. 77347

18 or/14-17 165100

19 10 and 13 and 18 9560

20 limit 19 to yr="2020 - 2022" 771

**Ovid MEDLINE(R**) ALL <1946 to January 29, 2021>

1 exp Artificial Intelligence/ 106412

2 ((artificial* or machine* or deep*) adj3 (intelligence or learning)).tw,kw. 55350

3 AI.ti,ab. 28603

4 exp Image Interpretation, Computer-Assisted/ 551508

5 computer* assist* diagnosis.tw,kw. 626

6 radiomic*.tw,kw. 3204

7 or/1-6 706556

8 exp Magnetic Resonance Imaging/ 465045

9 (Magnetic Resonance Imag* or MR-Imag* or MR Imag or MRI*).tw,kw. 435821

10 8 or 9 625065

11 exp Glioma/ 85314

12 glioma*.tw,kw. 60258

13 (glial adj2 (tumor or tumour)).tw,kw. 831

14 (glioblastoma* or astrocytoma* or astrocytic glioma* or astroglioma).tw,kw. 51784

15 or/11-14 115828

16 7 and 10 and 15 4493

17 limit 16 to yr="2020 - 2021" 260

**Cochrane CENTRAL (trials)**

ID Search Hits

#1 MeSH descriptor: [Artificial Intelligence] explode all trees 1040

#2 (artificial* OR machine* OR deep*) AND (intelligence OR learning) 3131

#3 AI 7937

#4 MeSH descriptor: [Image Processing, Computer-Assisted] explode all trees 3582

#5 computer* assist* diagnosis 6489

#6 radiomic* 210

#7 #1 OR #2 OR #3 OR #4 OR #5 OR #6 20843

#8 MeSH descriptor: [D008279] explode all trees 0

#9 Magnetic Resonance Imag* OR MR-Imag* OR MR Imag OR MRI* OR NMR 36332

#10 #8 OR #9 36332

#11 MeSH descriptor: [Glioma] explode all trees 1197

#12 glioma* 1792

#13 (glial AND (tumor OR tumour)) 70

#14 glioblastoma* OR astrocytoma* OR astrocytic glioma* OR astroglioma 2432

#15 #11 OR #12 OR #13 OR #14 3580

#16 #7 AND #10 AND #15 with Publication Year from 2020 to 2021, in Trials 2

**Web of Science**

# 13

235

#12

Indexes=SCI-EXPANDED, SSCI, A&HCI, CPCI-S, CPCI-SSH, BKCI-S, BKCI-SSH, ESCI, CCR-EXPANDED, IC Timespan=2020-2021

# 12

711

#11 AND #7 AND #6

Indexes=SCI-EXPANDED, SSCI, A&HCI, CPCI-S, CPCI-SSH, BKCI-S, BKCI-SSH, ESCI, CCR-EXPANDED, IC Timespan=All years

# 11

132,043

#10 OR #9 OR #8

Indexes=SCI-EXPANDED, SSCI, A&HCI, CPCI-S, CPCI-SSH, BKCI-S, BKCI-SSH, ESCI, CCR-EXPANDED, IC Timespan=All years

# 10

75,253

TS=(glioblastoma* or astrocytoma* or astrocytic glioma* or astroglioma)

Indexes=SCI-EXPANDED, SSCI, A&HCI, CPCI-S, CPCI-SSH, BKCI-S, BKCI-SSH, ESCI, CCR-EXPANDED, IC Timespan=All years

# 9

2,783

TS=(glial NEAR/2 (tumor* or tumour*))

Indexes=SCI-EXPANDED, SSCI, A&HCI, CPCI-S, CPCI-SSH, BKCI-S, BKCI-SSH, ESCI, CCR-EXPANDED, IC Timespan=All years

# 8

91,055

TS=(glioma*)

Indexes=SCI-EXPANDED, SSCI, A&HCI, CPCI-S, CPCI-SSH, BKCI-S, BKCI-SSH, ESCI, CCR-EXPANDED, IC Timespan=All years

# 7

1,046,719

TS=(Magnetic Resonance Imag* or MR-Imag* or MR Imag or MRI* or NMR)

Indexes=SCI-EXPANDED, SSCI, A&HCI, CPCI-S, CPCI-SSH, BKCI-S, BKCI-SSH, ESCI, CCR-EXPANDED, IC Timespan=All years

# 6

331,970

#5 OR #4 OR #3 OR #2 OR #1

Indexes=SCI-EXPANDED, SSCI, A&HCI, CPCI-S, CPCI-SSH, BKCI-S, BKCI-SSH, ESCI, CCR-EXPANDED, IC Timespan=All years

# 5

4,619

TS=(radiomic*)

Indexes=SCI-EXPANDED, SSCI, A&HCI, CPCI-S, CPCI-SSH, BKCI-S, BKCI-SSH, ESCI, CCR-EXPANDED, IC

# 4

6,845

TS=(computer* assist* diagnosis)

Indexes=SCI-EXPANDED, SSCI, A&HCI, CPCI-S, CPCI-SSH, BKCI-S, BKCI-SSH, ESCI, CCR-EXPANDED, IC Timespan=All years

# 3

51,251

AB=(AI)

Indexes=SCI-EXPANDED, SSCI, A&HCI, CPCI-S, CPCI-SSH, BKCI-S, BKCI-SSH, ESCI, CCR-EXPANDED, IC Timespan=All years

# 2

11,596

TI=(AI)

Indexes=SCI-EXPANDED, SSCI, A&HCI, CPCI-S, CPCI-SSH, BKCI-S, BKCI-SSH, ESCI, CCR-EXPANDED, IC Timespan=All years

# 1

276,118

TS=((artificial* or machine* or deep*) NEAR/3 (intelligence or learning))

Indexes=SCI-EXPANDED, SSCI, A&HCI, CPCI-S, CPCI-SSH, BKCI-S, BKCI-SSH, ESCI, CCR-EXPANDED, IC Timespan=All years

**Appendix A2. Article Screening.**

**Table A1. Agreement assessment between reviewers during article screening.**

| **Reviewer A** | **Reviewer B** | **A Yes, B Yes** | **A Yes, B No** | **A No, B Yes** | **A No, B No** | **Proportionate Agreement** | **Cohen's Kappa** |
| --- | --- | --- | --- | --- | --- | --- | --- |
| H.S. | M.S.A. | 601 | 113 | 168 | 8007 | 0.96839 | 0.7933 |
| H.S. | W.R.B. | 104 | 39 | 39 | 2062 | 0.96524 | 0.70871 |
| M.S.A. | W.R.B. | 79 | 1 | 27 | 254 | 0.92244 | 0.79859 |

**Appendix A3. Model Performance.**

**Table A2. Mean accuracy of studies grouped by dataset source (BraTS, TCIA, single center, multicenter (excluding BraTS and TCIA)).**

| **Dataset Source** | BraTS (n=27) | TCIA (n=12) | Single Center (n=43) | Multicenter (n=6) |
| --- | --- | --- | --- | --- |
| **Mean Accuracy** | 0.93±0.04 | 0.91±0.08 | 0.88±0.07 | 0.80±0.18 |

**Table A3. Mean accuracy of studies grouped by dataset size.**

| **Dataset Size** | ≤50  (n=16*) | 51-100  (n=9) | 101-200  (n=21) | 201-300  (n=20) | >300  (n=11) |
| --- | --- | --- | --- | --- | --- |
| **Mean Accuracy** | 0.91±0.07 | 0.83±0.12 | 0.88±0.10 | 0.90±0.05 | 0.89±0.14 |

*19 studies had dataset sizes ≤50 but only 16 reported accuracy values.

**Table A4. Mean accuracy of studies grouped by validation technique (external vs. internal).**

| **Validation Technique** | External Validation  (n=5) | Internal Validation  (n=66*) |
| --- | --- | --- |
| **Mean Accuracy** | 0.82±0.09 | 0.89±0.09 |

*68 studies internally validated their models but only 66 reported accuracy values.

**Table A5. Mean accuracy of studies grouped by glioma grade classification task.**

| **Glioma Grade Classification Task** | **Mean Accuracy** |
| --- | --- |
| 1 vs. 2 vs. 3 vs. 4 (n=3) | 0.92±0.06 |
| 1/2 vs. 3/4 (n=24) | 0.90±0.05 |
| 2 vs. 3 (n=7) | 0.87±0.08 |
| 2 vs. 3 vs. 4 (n=5) | 0.86±0.19 |
| 2 vs. 3/4 (n=8)* | 0.87±0.11 |
| 2/3 vs. 4 (n=11) | 0.89±0.07 |
| HGG vs. LGG (unclear) (n=13)** | 0.91±0.10 |

*9 studies performed 2 vs. 3/4 classifications but only 8 reported accuracy values.

**15 studies performed HGG vs. LGG classification but were unclear in their classification system. Only 13 reported accuracy values.

**Appendix A4. Study Characteristics.**

**Table A6. Characteristics of 85 studies reporting the highest accuracy results for their best performing models, including: glioma grade classification task, dataset source and size, ratio of high- to low-grade gliomas, validation technique, imaging sequences used in prediction, feature types used in prediction, best performing algorithm (based on accuracy results), performance metrics, and TRIPOD adherence rate (in percent). Testing or validation metrics are reported when available, otherwise training metrics are reported.**

| **Paper** | **Glioma Grade Classification Task** | **Dataset** | **HGG^a^/LGG^b^ Ratio** | **Validation Technique** | **Imaging Sequences** | **Features** | **Best Algorithm** | **Performance** | **TRIPOD^c^ Adherence** |
| --- | --- | --- | --- | --- | --- | --- | --- | --- | --- |
| Abd-Ellah et al. (2019) [1] | HGG vs. LGG vs. normal (unclear) | BraTS^d^ (n=1800 images) | 1:1  (150 HGG, 150 LGG in testing set) | Internal (Holdout, n=150 images) | Unclear | Deep learning extracted | CNN^e^ | Accuracy = 0.9744 Sensitivity = 0.97  Specificity = 0.98 | 22.2% |
| Abdolmaleki et al. (1997) [2] | 1/2 vs. 3/4 | Single center hospital (n=79 patients) | 4.1:1  (29 HGG, 7 LGG in testing set) | Unspecified | T1, T1CE^f^, T2 | Qualitative | ANN^g^ | Accuracy = 0.89 AUC^h^ = 0.9118 | 48.1% |
| Ali et al. (2019) [3] | 2 vs. 3/4 | BraTS (n=285 patients) | 2.8:1  (210 HGG, 75 LGG in total set) | Internal  (Holdout, 20% of dataset) | T1CE, T2, FLAIR | Deep learning extracted | DCGAN^i^ | Accuracy = 0.9204 | 40.7% |
| Alis et al. (2020) [4] | 1/2 vs. 3/4 | Single center hospital (n=181 patients) | 1.1:1  (32 HGG, 28 LGG in testing set) | Internal (10-fold cross-validation) | T1CE, (T2W)-FLAIR | Texture | ANN | Accuracy = 0.883 AUC = 0.92 Sensitivity = 0.875  Specificity = 0.892 | 55.6% |
| Al-Saffar and Yildirim (2020) [5] | HGG vs. LGG vs. normal (unclear) | TCIA^j^ (n=402 patients) | 1:1  (27 HGG, 27 LGG in testing set) | Internal (5-fold cross-validation) | FLAIR | Texture | Simplified RNN^k^ | Accuracy = 0.9491  Sensitivity = 0.9689  Specificity = 0.9637  PPV^l^ = 0.9637  NPV^m^ = 0.9429 | 44.4% |
| Al-Zurfi et al. (2018) [6] | 2/3 vs. 4 | TCIA (n=30 patients) | 1:2  (10 HGG, 20 LGG in training set) | Internal (Leave-one-out cross-validation) | T2 | Texture | BPNN incorporating 11 classifiers: DT^n^, LDA^o^, QDA^p^, SVM^q^ (SVML^r^, SVMQ^s^, SVMMG^t^), KNN^u^ (KNNF^v^, KNNM^w^, KNNCOS^x^, KNNCUB^y^, KNNW^z^) | Accuracy = 0.966  Sensitivity = 1.00  Specificity = 0.9 | 33.3% |
| Al-Zurfi et al. (2019) [7] | 1/2 vs. 3/4 | BraTS (n=30 patients) | 2:1  (20 HGG, 10 LGG in training set) | Internal (Leave-one-out cross-validation) | T2 | Texture | DT | Accuracy = 0.933  Sensitivity = 1.00  Specificity = 0.9 | 40.7% |
| Anaraki et al. (2019) [8] | HGG vs. LGG (unclear) | Multicenter hospital (n=unclear) | Unclear | Unspecified | T1CE | Deep learning extracted | CNN | Accuracy = 0.909 | 33.3% |
| BashirGonbadi and Khotanlou (2019) [9] | 1/2 vs. 3/4 | BraTS (n=285 patients) | 2.8:1  (210 HGG, 75 LGG in  total set) | Internal  (Holdout, 15% of dataset) | T1, T1CE, T2, FLAIR | Deep learning extracted | CNN | Accuracy = 0.9918 | 18.5% |
| Bisdas et al. (2018) [10] | 2 vs. 3 | Single center hospital (n=37 patients) | 1.1:1  (19 grade 2, 18 grade 3 in total set) | Internal (Leave-one-out cross-validation) | FLAIR, DWI^aa^ (DKI^ab^) | First-order, Texture | SVM | Accuracy = 0.781  AUC = 0.79  Sensitivity = 0.77  Specificity = 0.79 | 63.0% |
| Bonte et al. (2017) [11] | 2/3 vs. 4 | TCIA, REMBRANDT^ac^, Single center hospital (n=262 patients) | 1:2.4  (76 HGG, 186 LGG in  total set) | Internal (Leave-one-out cross-validation) | T1CE, FLAIR | First-order, Shape, Texture | RF^ad^ | Accuracy = 0.845 | 29.6% |
| Cao et al. (2020) [12] | 2/3 vs. 4 | TCIA (n=229 patients) | 1.3:1  (128 HGG, 101 LGG in  total set) | Internal (Holdout, 30% of dataset) | T1CE | Qualitative | RF | Accuracy = 0.852  AUC = 0.9 | 48.1% |
| Chen et al. (2020) [13] | HGG vs. LGG (unclear) | BraTS (n=285 patients) | 3:1  (43 HGG, 14 LGG in  testing set) | Unspecified | T1, T1CE, T2 | First-order,  Higher-order, Shape, Texture | SVM | Accuracy = 0.94  AUC = 0.99  Sensitivity = 0.94  Specificity = 0.95 | 40.7% |
| Chen et al. (2018) [14] | HGG vs. LGG (unclear) | BraTS (n=274 patients) | 4.1:1  (220 HGG, 54 LGG in  total set) | Internal (5-fold cross-validation) | T1, T1CE, T2, (T2W)-FLAIR | First-order, Shape, Texture | XGBoost^ae^ | Accuracy = 0.9127  Sensitivity = 0.9127  PPV = 0.9127  F1 score = 0.9064 | 44.4% |
| Chen et al. (2019) [15] | 1/2 vs. 3/4 | BraTS + GliomaHPPH2018^af^ (n=523 patients) | 2.4:1  (371 HGG, 152 LGG in  total set) | Internal (10-fold cross-validation) | T1C | First-order, Shape, Texture | DT, RF, BAG^ag^, BSA^ah^, NB^ai^, MLP^aj^, SVM, LR^ak^, KNN | Accuracy = 0.9413  AUC = 0.9786  Sensitivity = 0.9464  Specificity = 0.93 | 25.9% |
| Cheng et al. (2020) [16] | 1/2 vs. 3/4 | BraTS (n=350 patients) | 2.8:1  (48 HGG, 17 LGG in  validation set) | External (n=65 patients) | T1, T1CE, T2, (T2W)-FLAIR | First-order, Shape, Texture | RF | Accuracy = 0.94  AUC = 0.975  Sensitivity = 0.871  Specificity = 0.967 | 37.0% |
| Cho et al. (2018) [17] | HGG vs. LGG (unclear) | BraTS (n=285 patients) | 2.8:1  (210 HGG, 75 LGG in  total set) | Internal (5-fold cross-validation) | T1, T1CE, T2, FLAIR | First-order, Shape, Texture | LR, SVM, RF | Accuracy = 0.8947  AUC = 0.8765  Sensitivity = 0.9571  Specificity = 0.7200 | 44.4% |
| Christy et al. (1995) [18] | HGG vs. LGG (unclear) | Single center hospital (n=81 patients) | Unclear | Unspecified | T1, T1CE, T2 | Qualitative | Neural network | Accuracy = 0.61  Sensitivity = 0.64  Specificity = 0.56 | 59.3% |
| Cinarer et al. (2020) [19] | 2 vs. 3 | TCIA (n=121 patients) | 1:1.75  (44 HGG, 77 LGG in  total set) | Internal  (Holdout, n=21 patients) | T2, FLAIR | First-order, Texture | DNN^al^ | Accuracy = 0.9615  AUC = 0.9875  Sensitivity = 1.00  PPV = 0.9412  F1 score = 0.9697 | 44.4% |
| Citak-Er et al. (2018) [20] | 1/2 vs. 3/4 | Single center hospital (n=43 patients) | 1.9:1  (28 HGG, 15 LGG in  total set) | Internal (10-fold cross-validation) | T1, T1CE, T2, (T2W)-FLAIR, DWI (DTI^am^), MRS^an^ (TE^ao^ 144ms), PWI^ap^ | First-order, Qualitative,  Spectroscopic | SVM | Accuracy = 0.93  Sensitivity = 0.867  Specificity = 0.964 | 55.6% |
| Constantin et al. (2012) [21] | 2 vs. 3/4 (transformation of recurrent glioma) | Unclear (n=53 patients) | 1.4:1  (31 HGG, 22 LGG in  total set) | Internal (Leave-one-out cross-validation and bootstrapping) | MRS (TE 144ms) | Spectroscopic | LR | Accuracy = 0.63 | 37.0% |
| Cui et al. (2018) [22] | 1/2/3 vs. 4 | BraTS (n=80 patients) | 1:1  (40 HGG, 40 LGG in  total set) | Internal (Leave-one-out cross-validation) | T1, T1CE, T2, (T2W)-FLAIR | First-order,  Higher-order, Shape and size,  Texture | RF | Accuracy = 0.913  AUC = 0.956 | 37.0% |
| Cui et al. (2019) [23] | HGG vs. LGG (unclear) | BraTS (n=50 patients) | 1:1  (25 HGG, 25 LGG in  total set) | Internal (Leave-one-out cross-validation) | T1, T1CE, T2, (T2W)-FLAIR | First-order, Shape and size,  Texture | RF | Accuracy = 0.92  AUC = 0.962 | 37.0% |
| Dandil and Bicer (2020) [24] | 1/2 vs. 3 vs. 4 vs. meningioma | INTERPRET^aq^ (n=179 patients) | Unclear | Unspecified | MRS (TE 20ms, 136ms) | First-order, Shape and size,  Texture | LSTM^ar^ (neural network) | Accuracy = 0.982  AUC = 0.9936  Sensitivity = 1.00  Specificity = 0.9753 | 48.1% |
| De Looze et al. (2018) [25] | HGG vs. LGG (unclear) | Single center hospital (n=381 patients) | Unclear | Internal (5-fold cross-validation) | T1, T1CE, T2, FLAIR, DWI | Qualitative | RF | Accuracy = 0.99  AUC = 0.99  Sensitivity = 1.00  Specificity = 0.92 | 63.0% |
| Decuyper et al. (2018) [26] | 2/3 vs. 4 | BraTS (n=285 patients) | 2.8:1  (210 HGG, 75 LGG in  total set) | Internal (5-fold cross-validation) | T1CE | First-order, Shape and size,  Texture | RF | Accuracy = 0.896  AUC = 0.964  Sensitivity = 0.899  Specificity = 0.888 | 25.9% |
| Devos et al. (2005) [27] | HGG vs. LGG (unclear) | Single center hospital (n=21 patients) | Unclear (10 grade 2, 4 grade 3, and 7 grade 4 gliomas in total set) | Unspecified | T1, T1CE, T2, MRS (TE 20ms) | Spectroscopic | LS-SVM^as^ | AUC = 0.992 | 44.4% |
| Dong et al. (2019) [28] | 1 vs. 4 | Multicenter hospital (n=66 patients) | 1.1:1  (35 HGG, 31 LGG in  total set) | Internal  (Holdout, n=22 patients) | T1, T1CE, T2 | Texture | DT | Accuracy = 0.86  Sensitivity = 0.80  Specificity = 0.91 | 55.6% |
| Emblem et al. (2008) [29] | 1/2 vs. 3/4 | Single center hospital (n=86 patients) | 1.4:1  (19 HGG, 14 LGG in  testing set) | Internal (10-fold cross-validation) | T1, T1CE, T2, PWI (DSC^at^) | First-order | *ν*-SVM^au^ | Accuracy = 0.8396  Sensitivity = 0.76  Specificity = 0.82 | 48.1% |
| Gao et al. (2020) [30] | 1/2 vs. 3/4 | Single center hospital (n=369 patients) | 1.5:1  (222 HGG, 147 LGG in  total set) | Internal (Cross-validation, *k* fold validation) | T1CE | Shape, First-order | RF | Accuracy = 0.81  AUC = 0.79  Sensitivity = 0.63  Specificity = 0.89  F1 score = 0.67 | 51.9% |
| Gates et al. (2020) [31] | 2 vs. 3 vs. 4 | Single center hospital (n=23 patients) | 1:1.3:1  7 grade 2, 9 grade 3, 7 grade 4 in total set | Internal (5-fold cross-validation) | T2, DWI (DTI), PWI (DSC/DCE) | Hemodynamic,  Texture | RF | Accuracy = 0.952  AUC = 0.985 | 63.0% |
| Ge et al. (2018) [32] | 1/2 vs. 3/4 | BraTS (n=285 patients) | 2.8:1  (210 HGG, 75 LGG in  total set) | Internal  (Holdout, n=57 patients) | T1CE, T2, FLAIR | Deep learning extracted | CNN | Accuracy = 0.9087 | 37.0% |
| Ge et al. (2020) [33] | 2 vs. 3/4 | BraTS (n=285 patients) | 2.8:1  (210 HGG, 75 LGG in  total set) | Internal  (Holdout, 30% of dataset) | T1, T1CE, T2, FLAIR | Deep learning extracted | CNN | Accuracy = 0.907  Sensitivity = 0.8435  Specificity = 0.9301 | 40.7% |
| Ge et al. (2018) [34] | 2 vs. 3/4 | BraTS (n=285 patients) | 2.8:1  (210 HGG, 75 LGG in  total set) | Internal  (n=57 patients) | T1CE | Deep learning extracted | CNN | Accuracy = 0.8947 | 37.0% |
| Gutta et al. (2021) [35] | 1 vs. 2 vs. 3 vs. 4 | Single center hospital (n=237 patients) | 1:3.5:2.7:6.8  (17 grade 1, 59 grade 2, 46 grade 3, 115 grade 4 in  total set) | Internal  (Holdout, 15% of dataset) | T1, T1CE, T2, (T2W)-FLAIR | Deep learning extracted | CNN | Accuracy = 0.87 | 48.1% |
| Haubold et al. (2020) [36] | HGG vs. LGG (unclear) | Single center hospital (n=34 patients) | Unclear | Internal (5-fold cross-validation) | T1CE, PET-MRI (18F-FET^av^), SWI^aw^ | First-order,  Shape,  Texture | RF | AUC = 0.852  Sensitivity = 0.831  Specificity = 0.796 | 51.9% |
| Hedyehzadeh et al. (2020) [37] | 2/3 vs. 4 | TCIA (n=461 patients) | 1.3:1  (262 HGG, 199 LGG in  total set) | Internal (4-fold cross-validation | T1, T1CE, T2, FLAIR | Texture | SVM | Accuracy = 1.00  Sensitivity = 1.00  Specificity = 1.00 | 25.9% |
| Hsieh et al. (2017) [38] | 2/3 vs. 4 | TCIA (n=105 patients) | 1:2.1  (34 HGG, 71 LGG in  total set) | Internal (Leave-one-out cross-validation) | T1CE | First-order,  Texture | Neuroradiologist + CAD^ax^ (unspecified) | Accuracy = 0.81  AUC = 0.9  Sensitivity = 0.76  Specificity = 0.83  PPV = 0.68  NPV = 0.88 | 48.1% |
| Inano et al. (2014) [39] | 2 vs. 3/4 | Single center hospital (n=33 patients) | 1.4:1  (19 HGG, 14 LGG in  total set) | Internal (Leave-one-out cross-validation) | DWI (DTI) | First-order | SVM | Accuracy = 0.804  AUC = 0.912  Sensitivity = 0.848  Specificity = 0.745 | 59.3% |
| Jeong et al. (2019) [40] | HGG vs. LGG (unclear) | Single center hospital (n=25 patients) | 1:1.1  (12 HGG, 13 LGG in  total set) | Internal (Leave-one-out cross-validation) | (T2W)-FLAIR, PWI (DSC) | Texture | RF | Accuracy = 0.902  AUC = 0.938 | 51.9% |
| Ji et al. (2019) [41] | 2 vs. 3/4 | Single center hospital (n=30 patients) | 1.1:1  (16 HGG, 14 LGG in  total set) | Internal (5-fold cross-validation) | PWI (DSC) | Hemodynamic | LR | Accuracy = 0.867  AUC = 0.77 | 48.1% |
| Kaur et al. (2018) [42] | HGG vs. LGG (unclear) | Single center hospital (n=50 patients) | 1:1.2  (23 HGG, 27 LGG in  total set) | Internal (5-fold cross-validation) | MRS (TE 135ms) | Spectroscopic | KNN | Accuracy = 0.94  Sensitivity = 0.96  Specificity = 0.91 | 40.7% |
| Khawaldeh et al. (2018) [43] | 2 vs. 3/4 vs. normal | TCIA (n=130 patients) | 1:1.1  (252 HGG images, 267 LGG images in  validation set) | Internal  (Holdout, n=674 images) | FLAIR | Deep learning extracted | AlexNet | Accuracy = 0.9116  Sensitivity = 0.9225  PPV = 0.9179  F1 Score 0.9205 | 40.7% |
| Koyuncu et al. (2020) [44] | 1/2 vs. 3/4 | BraTS (n=285 patients) | 2.8:1  (210 HGG, 75 LGG in  total set) | Internal (2-fold cross-validation) | T1, T1CE, T2, FLAIR | First-order | GM-CPSO-NN^ay^ | Accuracy = 0.9018  AUC = 0.8562  Sensitivity = 0.9524  Specificity = 0.76 | 37.0% |
| Kumar et al. (2020) [45] | 1/2 vs. 3/4 | BraTS (n=285 patients) | 2.8:1  (210 HGG, 75 LGG in  total set) | Internal (5-fold cross-validation) | T1, T1CE, T2, (T2W)-FLAIR | First-order,  Shape,  Texture | RF | Accuracy = 0.9754  AUC = 0.9748  Sensitivity = 0.9762  Specificity = 0.9733  F1 Score = 0.983 | 33.3% |
| Lo et al. (2019) [46] | 2 vs. 3 vs. 4 | TCIA (n=130 patients) | 1:1.4:1.9  (30 grade 2, 43 grade 3 and 57 grade 4 in total set) | Internal (10-fold cross-validation) | T1CE | Deep learning extracted | DCNN^az^ | Accuracy = 0.979  AUC = 0.9991 | 40.7% |
| Ma et al. (2020) [47] | 1/2 vs. 3/4 | Single center hospital (n=662 patients) | 1:1.6  (252 HGG, 410 LGG in  total set) | Internal (Holdout, 20% of dataset) | T1CE | First-order,  Shape,  Texture | XGBoost | Accuracy = 0.83  AUC = 0.86  Sensitivity = 0.86  Specificity = 0.81  PPV = 0.84 (LGG), 0.83 (HGG)  F1 Score = 0.83 (LGG), 0.84 (HGG) | 51.9% |
| Muneer et al. (2019) [48] | 1 vs. 2 vs. 3 vs. 4 | Single center hospital (n=20 patients) | 1.3:1.6:1:1.5  (39 grade 1, 51 grade 2, 31 grade 3, 47 grade 4 images in testing set) | Internal (Holdout, 30% of dataset) | T2 | Deep learning extracted | VGG19^ba^ | Accuracy = 0.9825  Sensitivity = 0.9272  Specificity = 0.9813  PPV = 0.9471  F1 Score = 0.9371 | 40.7% |
| Mzoughi et al. (2020) [49] | HGG vs. LGG (unclear) | BraTS (n=351 patients) | 2.8:1  (209 HGG, 75 LGG in  training set) | Unclear | T1CE | Deep learning extracted | CNN | Accuracy = 0.9649 | 40.7% |
| Nakamoto et al. (2019) [50] | 3 vs 4 | TCIA (n=157 patients) | 1:2  (22 grade 3 and 45 grade 4 in validation set) | External  (n=67 patients) | T1CE, T2 | First-order,  Shape and size,  Texture | SVM | Accuracy = 0.746  AUC = 0.731  Sensitivity = 0.844  Specificity = 0.545 | 37.0% |
| Naser and Deen (2020) [51] | 2 vs. 3 | TCIA (n=110 patients) | 1:1  (50 grade 2 and 48 grade 3 in total set) | Internal (Cross-validation) | T1, T1CE, FLAIR | Deep learning extracted | VGG16^bb^ | Accuracy = 0.89  Sensitivity = 0.87  Specificity = 0.92 | 48.1% |
| Park et al. (2019) [52] | 2 vs. 3 | Single center hospital (n=204 patients) | Unclear | External  (n=99 patients) | T1CE, T2, FLAIR | First-order,  Shape,  Texture | Elastic Net | Accuracy = 0.794  AUC = 0.85  Sensitivity = 0.929  Specificity = 0.7 | 59.3% |
| Polly et al. (2018) [53] | HGG vs. LGG (unclear) | BraTS (n=160 images) | 1:1  (50 HGG, 50 LGG in  testing set) | Unspecified | T2 | First-order,  Shape,  Texture | SVM | Accuracy = 0.99  Sensitivity = 1.00  Specificity = 0.9803 | 33.3% |
| Qi et al. (2019) [54] | 2/3 vs. 4 | Single center hospital (n=112 patients) | 1:3.7  (8 HGG, 30 LGG in  validation set) | External  (n=38 patients) | T2, MRS (TE 105 ms) | Spectroscopic | SVM | Accuracy = 0.7838  AUC = 0.825  Sensitivity = 0.742  Specificity = 0.814 | 40.7% |
| Rajasree et al. (2021) [55] | 1/2 vs. 3/4 | BraTS (n=274 patients) | 4.1:1  (220 HGG, 54 LGG in  total set) | Internal  (5-fold cross-validation) | T1, T1CE, T2, FLAIR | Deep learning extracted | MSMCNN^bc^ | Accuracy = 0.9636  Sensitivity = 0.9214  PPV = 0.9161 | 33.3% |
| Ranjith et al. (2015) [56] | 2 vs. 3/4 | Single center hospital (n=28 patients) | 1:1.3  (12 HGG, 16 LGG in  total set) | Internal  (5-fold cross-validation) | MRS (TE 135 ms) | Spectroscopic | RF | AUC = 0.911  Sensitivity = 0.806  Specificity = 0.857  PPV = 0.806  NPV = 0.857 | 44.4% |
| Reza et al. (2019) [57] | 1/2 vs. 3/4 | BraTS (n=285 patients) | 2.8:1  (210HGG, 75 LGG in  total set) | Internal  (10-fold cross-validation) | T1CE, T2, FLAIR | First-order,  Shape,  Texture | RF | Accuracy = 0.88  AUC = 0.88  Sensitivity = 0.98  Specificity = 0.62 | 44.4% |
| Sajjad et al. (2019) [58] | 1 vs. 2 vs. 3 vs. 4 | Multicenter hospital (n=121 images) | 1.4:1.3:1:1.1  (36 grade 1, 32 grade 2, 25 grade 3, 28 grade 4 in total set) | Internal  (Holdout, 25% of dataset) | Unclear | Deep learning extracted | CNN | Accuracy = 0.9067 | 29.6% |
| Saxena et al. (2019) [59] | oligodendroglioma vs. astrocytoma vs. GBM^bd^ (individual grades unclear) | REMBRANDT (n=130 patients) | Unclear | Internal  (5-fold cross-validation) | Unclear | Deep learning extracted, Shape,  Texture | CNN | Accuracy = 0.5667 | 29.6% |
| Sengupta et al. (2019) [60] | 2 vs. 3 | Single center hospital (n=66 patients) | 2.2:1  (9 HGG, 4 LGG in validation set) | Internal  (Holdout, n=13 patients) | T1,T2, FLAIR, PWI (DCE) | Hemodynamic | SVM | Accuracy = 0.963  AUC = 0.98  Sensitivity = 0.92  Specificity = 1.00 | 51.9% |
| Sharif et al. (2020) [61] | HGG vs. LGG ( unclear) | BraTS (n=30 patients) | 2.3:1  (7 HGG, 3 LGG in testing set) | Internal (Holdout, 10-fold cross-validation) | T1, T1CE, T2, FLAIR | Deep learning extracted | CNN | Accuracy = 0.987 | 33.3% |
| Subashini et al. (2016) [62] | 1/2 vs. 3/4 | Single center hospital (n=200 patients) | 1:1  (18 HGG, 18 LGG in testing set) | Unspecified | T2 | First-order,  Shape,  Texture | LVQ^be^ | Accuracy = 0.9167 | 33.3% |
| Sudre et al. (2020) [63] | 2 vs. 3 vs. 4 | Multicenter hospital (n=333 patients) | 1.4:1:2.1  (101 grade 2, 74 grade 3, 158 grade 4 in testing set) | Internal  (2-fold cross-validation) | T1, T2, FLAIR, PWI (DSC) | First-order,  Shape,  Texture | RF | Accuracy = 0.53 | 59.3% |
| Sun et al. (2019) [64] | 1/2 vs. 4 | BraTS (n=285 patients) | 2.8:1  (210 HGG, 75 LGG in  total set) | Internal  (10-fold cross-validation) | T1, T1CE, T2, (T2W)-FLAIR | First-order,  Shape,  Texture | SVM | Accuracy = 0.953  AUC = 0.981 | 37.0% |
| Takahashi et al. (2019) [65] | 2/3 vs. 4 | Single center hospital (n=55 patients) | 2.9:1  (41 HGG, 14 LGG in total set) | Internal (Cross-validation) | DWI (DKI, DTI) | First-order, Higher-order,  Shape,  Texture | SVM | Accuracy = 0.91  AUC = 0.93 | 51.9% |
| Tian et al. (2018) [66] | 2 vs. 3/4 | Single center hospital (n=153 patients) | 2.6:1  (111 HGG, 42 LGG in total set) | Internal  (10-fold cross-validation) | T1, T1CE, T2, DWI, PWI (3D-ASL^bf^) | Texture | SVM | Accuracy = 0.981  AUC = 0.992  Sensitivity = 0.987  Specificity = 0.974 | 63.0% |
| Tian et al. (2019) [67] | 3 vs. 4 | Single center hospital (n=123 patients) | 1:1.7  (46 grade 3 and 76 grade 4 in total set) | Internal  (Holdout, 20% of dataset) | T1CE | First-order,  Shape,  Texture | LDA | Accuracy = 0.968  AUC = 0.974  Sensitivity = 0.927  Specificity = 0.989 | 59.3% |
| Ural et al. (2020) [68] | HGG vs. LGG vs. GBM vs. meningioma vs. ischemic stroke vs. hemorrhagic stroke (individual grades unclear) | BraTS (n=300 images) | Unclear | Unspecified | T1, T1CE, T2, FLAIR | First-order,  Texture | SVM | Accuracy = 0.927  Sensitivity = 0.968  Specificity = 0.98 | 22.2% |
| Vamvakas et al. (2019) [69] | 1/2 vs. 3/4 | Single center hospital (n=40 patients) | 1:1  (40 HGG, 40 LGG in total set) | Internal (Leave-one-out cross-validation) | T1CE, T2, FLAIR, DWI (DTI), MRS (TE 35ms, 144ms), PWI (DSC) | Spectroscopic,  Texture | SVM | Accuracy = 0.955  AUC = 0.955  Sensitivity = 0.95  Specificity = 0.96 | 40.7% |
| Van Cauter et al. (2014) [70] | 1/2 vs. 3/4 | Single center hospital (n=54 patients) | 1.1:1  (10 HGG, 9 LGG in validation set) | External  (n=19 patients) | DWI (DKI), MRS (TE 80ms), PWI (DSC) | First-order, Hemodynamic, Spectroscopic | LDA | Accuracy = 0.83  Sensitivity = 0.78  Specificity = 0.91  PPV = 0.93  NPV = 0.73 | 59.3% |
| Wiestler et al. (2016) [71] | 2/3 vs. 4 | Single center hospital (n=37 patients) | 1.1:1  (27 HGG, 10 LGG in total set) | Internal  (5-fold cross-validation) | T1CE, T2, FLAIR, PWI (DSC) | First-order | RF | Accuracy = 0.918  AUC = 0.944  Sensitivity = 0.8889  Specificity = 1.00  PPV = 1.00  NPV = 0.7692 | 37.0% |
| Wu et al. (2015) [72] | 1/2 vs. 3/4 | Single center hospital (n=35 patients) | 1:1.1  (17 HGG, 18 LGG in total set) | Unspecified | T1, fMRI^bg^ | Hemodynamic | SVM | Accuracy = 0.89  AUC = 0.89  Sensitivity = 0.82  Specificity = 0.96 | 48.1% |
| Wu et al. (2019) [73] | 1/2 vs. 3/4 | BraTS + GliomaHPPH2018 (n=518 patients) | 2.4:1  (367 HGG, 151 LGG in total set) | Internal  (4-fold cross-validation) | T1, T1CE, T2, FLAIR | First-order,  Higher-order,  Texture | MMEDT^bh^ (DT) | Accuracy = 0.8743  AUC = 0.9084  Sensitivity = 0.8791  Specificity = 0.8739  PPV = 0.9258  NPV = 0.7651 | 44.4% |
| Wu et al. (2018) [74] | HGG vs. LGG (unclear) | Single center hospital (n=161 patients) | 2.4:1  (109 HGG, 54 LGG in total set) | Internal  (10-fold cross-validation) | T1 | First-order,  Shape,  Texture | Linear Regression | Accuracy = 0.913  AUC = 0.9638  Sensitivity = 0.9357  Specificity = 0.8653 | 51.9% |
| Yang et al. (2018) [75] | 2/3 vs. 4 | Single center hospital (n=113 patients) | 1.2:1  (61 HGG, 52 LGG in total set) | Internal  (5-fold cross-validation) | T1CE | Deep learning extracted | GoogLeNet | Accuracy = 0.847  AUC = 0.966 | 48.1% |
| Yang et al. (2019) [76] | 2 vs. 3 vs. 4 | Single center hospital (n=117 patients) | 1:1.2:2.5  (25 grade 2, 29 grade 3, 63 grade 4 in total set) | Internal  (10-fold cross-validation) | T1, T1CE, (T2W)-FLAIR, DWI, PWI (DCE, 3D-ASL) | First-order,  Texture | SVM | Accuracy = 0.875  AUC = 0.971 | 59.3% |
| Ye et al. (2002) [77] | 1/2 vs. 3/4 | Single center hospital (n=280 patients) | 1:1.5  (111 HGG, 169 LGG in total set) | Internal  (10-fold cross-validation) | T1, T2 | Clinical,  First-order, Qualitative | MLP | Accuracy = 0.846 | 66.7% |
| Zhan et al. (2017) [78] | 1/2 vs. 3/4 | BraTS (n=274 patients) | 4.1:1  (220 HGG, 54 LGG in validation set) | Unspecified | T1, T1CE, T2, FLAIR | First-order,  Shape,  Texture | KNN | Accuracy = 0.8759 | 29.6% |
| Zhang et al. (2017) [79] | 2 vs. 3 vs. 4 | Single center hospital (n=117 patients) | 1:1.2:2.5  (25 grade 2, 29 grade 3, 63 grade 4 in total set) | Internal (Leave-one-out cross-validation) | T1CE, FLAIR, DWI, PWI (DCE, 3D-ASL) | First-order, Texture | IBk^bi^ | Accuracy = 0.961  AUC = 0.971 | 59.3% |
| Zhang et al. (2019) [80] | 2 vs. 3 | Single center hospital (n=101 patients) | 1:1  (50 HGG, 51 LGG in total set) | Internal  (Holdout, 20% of dataset) | T1CE | Texture | RF | Accuracy = 0.9  AUC = 0.904  Sensitivity = 0.971  Specificity = 0.833 | 55.6% |
| Zhang et al. (2020) [81] | 2 vs. 3/4 | Single center hospital (n=108 patients) | 1.5:1  (65 HGG, 43 LGG in total set) | Internal (Leave-one-out cross-validation) | DWI (DTI) | Deep learning extracted, Shape, Texture | SVM | Accuracy = 0.94  AUC = 0.93  Sensitivity = 0.98  Specificity = 0.86 | 55.6% |
| Zhao et al. (2020) [82] | 2 vs. 3 | Single center hospital (n=36 patients) | 1.1:1  (19 grade 2, 17 grade 3 in total set) | Internal  (5-fold cross-validation) | T1CE, FLAIR | First-order, Shape,  Texture | RF | Accuracy = 0.781  AUC = 0.861  Sensitivity = 0.778  Specificity = 0.783 | 55.6% |
| Zhuge et al. (2020) [83] | 2/3 vs. 4 | BraTS + TCIA (n=315 patients) | Unclear | Internal  (Holdout, 20% of dataset) | T1, T1CE, T2, FLAIR | Deep learning extracted | 3DConvNet^bj^ | Accuracy = 0.971  Sensitivity = 0.947  Specificity = 0.968 | 44.4% |
| Zollner et al. (2010) [84] | 1/2 vs. 3/4 | Single center hospital (n=101 patients) | 1.7:1  (63 HGG, 38 LGG in total set) | Internal  (10-fold cross-validation) | T1, T1CE, T2, FLAIR, PWI (DSC) | First-order | SVM | Accuracy = 0.87  AUC = 0.87  Sensitivity = 0.83  Specificity = 0.91 | 55.6% |
| Zollner et al. (2012) [85] | 1/2 vs. 3/4 | Single center hospital (n=101 patients) | 1.7:1  (63 HGG, 38 LGG in total set) | Internal  (10-fold cross-validation) | PWI (DSC) | Clinical,  First-order | SVM | Accuracy = 0.87  Sensitivity = 0.83  Specificity = 0.91 | 44.4% |

^a^HGG = High-Grade Glioma

^b^LGG = Low-Grade Glioma

^c^TRIPOD = The Transparent Reporting of a multivariable prediction model for Individual Prognosis or Diagnosis Statement

^d^BraTS = Brain Tumor Segmentation Challenge

^e^CNN = Convolutional Neural Network

^f^T1CE = T1-Weighted Contrast-Enhanced

^g^ANN = Artificial Neural Network

^h^AUC = Area Under the Receiver Operating Characteristic Curve

^i^DCGAN = Deep Convolutional Generative Adversarial Network

^j^TCIA = The Cancer Imaging Archive

^k^RNN = Recurrent Neural Network

^l^PPV = Positive Predictive Value

^m^NPV = Negative Predictive Value

^n^DT = Decision Tree

^o^LDA = Linear Discriminant Analysis

^p^QDA = Quadratic Discriminant Analysis

^q^SVM = Support Vector Machine

^r^SVML = Support Vector Machine (kernel function is linear)

^s^SVMQ = Support Vector Machine (kernel function is quadratic)

^t^SVMMG = Support Vector Machine (kernel function is medium Gaussian)

^u^KNN = K-Nearest Neighbors

^v^KNNF = K-Nearest Neighbors (number of k-neighbors is 1; distance metric is Euclidean; distance weight is identical)

^w^KNNM = K-Nearest Neighbors (number of k-neighbors is 10; distance metric is Euclidean; distance weight is identical)

^x^KNNCOS = K-Nearest Neighbors (number of k-neighbors is 10; distance metric is cosine; distance weight is identical)

^y^KNNCUB = K-Nearest Neighbors (number of k-neighbors is 10; distance metric is cosine, distance weight is identical)

^z^KNNW = K-Nearest Neighbors (number of k-neighbors is 10; distance metric is Euclidean; distance weight is square inverse)

^aa^DWI = Diffusion-Weighted Imaging

^ab^DKI = Diffusion Kurtosis Imaging

^ac^REMBRANDT = The Repository of Molecular Brain Neoplasia Data (citation?)

^ad^RF = Random Forest

^ae^XGBoost = eXtreme Gradient Boosting

^af^GliomaHPPH2018 = Henan Provincial People's Hospital (data set)

^ag^BAG = Bagging

^ah^BSA = Binary Search Tree

^ai^NB = Naive Bayes

^aj^MLP = Multilayer Perceptron

^ak^LR = Logistic Regression

^al^DNN = Deep Neural Network

^am^DTI = Diffusion Tensor Imaging

^an^MRS = Magnetic Resonance Spectroscopy

^ao^TE = Time of Echo

^ap^PWI = Perfusion-Weighted Imaging

^aq^INTERPRET = International Network for Pattern Recognition of Tumours Using Magnetic Resonance

^ar^LSTM = Long Short-Term Memory

^as^LS-SVM = Least Squares Support Vector Machine

^at^DSC = Dynamic Susceptibility Contrast

^au^ν-SVM = Nu-Support Vector Machine

^av^18F-FET = 18F-Fluoro-Ethyl-Tyrosine

^aw^SWI = Susceptibility Weighted Imaging

^ax^CAD = Computer-Aided Diagnosis

^ay^GM-CPSO-NN = Gauss-Map-Based Chaotic Particle-Swarm Optimization Neural Network

^az^DCNN = Deep Convolutional Neural Network

^bb^VGG19 = Visual Geometry Group (19-Layer Convolutional Neural Network)

^bb^VGG16 = Visual Geometry Group (16-Layer Convolutional Neural Network)

^bc^MSMCNN = Multiscale Multimodal Convolutional Neural Network

^bd^GBM = Glioblastoma

^be^LVQ = Learning Vector Quantization

^bf^3D-ASL = 3D Arterial Spin Labeling

^bg^fMRI = Functional Magnetic Resonance Imaging

^bh^MMEDT = Multi-Maximum Entropy Discrimination Decision Tree

^bi^IBk - Instance-Based Learner

^bj^3DConvNet = 3D Volumetric Convolutional Neural Network

**Table A7. Feature Types and Definitions.**

| **Feature Type** | **Definition** |
| --- | --- |
| Clinical | Features describing clinical information about the patient, e.g., gender and age. |
| Deep learning extracted | Features derived from pre-trained deep neural networks. |
| First-order | Features creating a three-dimensional (3D) histogram out of tumor volume characteristics, from which common statistics can be calculated, e.g., mean, median, range, skewness, kurtosis. [86] |
| Hemodynamic | Features describing hemodynamic signal profiles and heterogeneity, e.g., MR perfusion extracted features. |
| Higher-order | Features used to identify repetitive or non-repetitive patterns of image data, suppress noise, or highlight details. [86] |
| Qualitative | Features describing the baseline visual characteristics of the tumor on clinical MRI using controlled vocabulary, e.g., VASARI features (tumor location, side of lesion center, enhancement quality, etc.). |
| Shape and Size | Features describing the statistical inter-relationships between neighboring voxels, e.g., total volume or surface area, surface-to-volume ratio, tumor compactness, sphericity, etc. [86] |
| Spectroscopic | Features derived from MR Spectroscopy, e.g., peak integral values of choline, n-acetyl aspartate (NAA) and creatinine metabolites. |
| Texture | Otherwise known as second-order statistics or co-occurrence matrix features, they can be used for texture classification, e.g., contrast, correlation, dissimilarity, maximum probability, grey level run length features, etc. [86] |

**REFERENCES**

1. Abd-Ellah MK, Awad AI, Hamed HEA, Khalaf AAM. Parallel Deep CNN Structure for Glioma Detection and Classification via Brain MRI Images. Int C Microelectron. 2019:304-7. PubMed PMID: WOS:000555677600071.
2. Abdolmaleki P, Mihara F, Masuda K, Buadu LD. Neural networks analysis of astrocytic gliomas from MRI appearances. Cancer Lett. 1997;118(1):69-78. doi: 10.1016/s0304-3835(97)00233-4. PubMed PMID: 9310262.
3. Ali MB, Gu IYH, Jakola AS. Multi-stream Convolutional Autoencoder and 2D Generative Adversarial Network for Glioma Classification. Lect Notes Comput Sc. 2019;11678:234-45. doi: 10.1007/978-3-030-29888-3_19. PubMed PMID: WOS:000558153800019.
4. Alis D, Bagcilar O, Senli YD, Isler C, Yergin M, Kocer N, et al. The diagnostic value of quantitative texture analysis of conventional MRI sequences using artificial neural networks in grading gliomas. Clin Radiol. 2020;75(5):351-7. doi: 10.1016/j.crad.2019.12.008. PubMed PMID: 31973941.
5. Al-Saffar ZA, Yildirim T. A Novel Approach to Improving Brain Image Classification Using Mutual Information-Accelerated Singular Value Decomposition. Ieee Access. 2020;8:52575-87. doi: 10.1109/Access.2020.2980728. PubMed PMID: WOS:000524748500117.
6. Al-Zurfi A, Meziane F, Aspin R. Automated Glioma Grading based on an Efficient Ensemble Design of a Multiple Classifier System using Deep Iteration Neural Networks Matrix. 2018 24th Ieee International Conference on Automation and Computing (Icac' 18). 2018:38-43. PubMed PMID: WOS:000491276900007.
7. Al-Zurfi AN, Meziane F, Aspin R. A Computer-aided Diagnosis System for Glioma Grading using Three Dimensional Texture Analysis and Machine Learning in MRI Brain Tumour. 2019 3rd International Conference on Bio-Engineering for Smart Technologies (Biosmart). 2019. PubMed PMID: WOS:000492844100009.
8. Anaraki AK, Ayati M, Kazemi F. Magnetic resonance imaging-based brain tumor grades classification and grading via convolutional neural networks and genetic algorithms. Biocybern Biomed Eng. 2019;39(1):63-74. doi: 10.1016/j.bbe.2018.10.004. PubMed PMID: WOS:000462350100006.
9. BashirGonbadi F, Khotanlou H. Glioma Brain Tumors Diagnosis and Classification in MR Images based on Convolutional Neural Networks. 2019 9th International Conference on Computer and Knowledge Engineering (Iccke 2019). 2019:375-9. PubMed PMID: WOS:000540216700061.
10. Bisdas S, Shen H, Thust S, Katsaros V, Stranjalis G, Boskos C, et al. Texture analysis- and support vector machine-assisted diffusional kurtosis imaging may allow in vivo gliomas grading and IDH-mutation status prediction: a preliminary study. Sci Rep. 2018;8(1):6108. doi: 10.1038/s41598-018-24438-4. PubMed PMID: 29666413; PubMed Central PMCID: PMC5904150.
11. Bonte S, Goethals I, Van Holen R. Individual Prediction of Brain Tumor Histological Grading Using Radiomics on Structural MRI. Ieee Nucl Sci Conf R. 2017. PubMed PMID: WOS:000455836200202.
12. Cao H, Erson-Omay EZ, Li XJ, Gunel M, Moliterno J, Fulbright RK. A quantitative model based on clinically relevant MRI features differentiates lower grade gliomas and glioblastoma. European Radiology. 2020;30(6):3073-82. doi: 10.1007/s00330-019-06632-8. PubMed PMID: WOS:000515889600004.
13. Chen QJ, Wang LH, Wang L, Deng ZY, Zhang J, Zhu YM. Glioma Grade Prediction Using Wavelet Scattering-Based Radiomics. Ieee Access. 2020;8:106564-75. doi: 10.1109/Access.2020.3000895. PubMed PMID: WOS:000544040800009.
14. Chen W, Liu BQ, Peng ST, Sun JW, Qiao X. Computer-Aided Grading of Gliomas Combining Automatic Segmentation and Radiomics. International Journal of Biomedical Imaging. 2018;2018. doi: Artn 2512037 10.1155/2018/2512037. PubMed PMID: WOS:000433223800001.
15. Chen X, Wu Y, Zhao G, Wang M, Gao W, Zhang Q, et al. Automatic Histogram Specification for Glioma Grading Using Multicenter Data. J Healthc Eng. 2019;2019:9414937. doi: 10.1155/2019/9414937. PubMed PMID: 31934325; PubMed Central PMCID: PMC6942805.
16. Cheng J, Liu J, Yue H, Bai H, Pan Y, Wang J. Prediction of Glioma Grade using Intratumoral and Peritumoral Radiomic Features from Multiparametric MRI Images. IEEE/ACM Trans Comput Biol Bioinform. 2020;PP. doi: 10.1109/TCBB.2020.3033538. PubMed PMID: 33104503.
17. Cho HH, Lee SH, Kim J, Park H. Classification of the glioma grading using radiomics analysis. PeerJ. 2018;6:e5982. doi: 10.7717/peerj.5982. PubMed PMID: 30498643; PubMed Central PMCID: PMC6252243.
18. Christy PS, Tervonen O, Scheithauer BW, Forbes GS. Use of a Neural-Network and a Multiple-Regression Model to Predict Histologic Grade of Astrocytoma from Mri Appearances. Neuroradiology. 1995;37(2):89-93. PubMed PMID: WOS:A1995QF87200001.
19. Cinarer G, Emiroglu BG, Yurttakal AH. Prediction of Glioma Grades Using Deep Learning with Wavelet Radiomic Features. Appl Sci-Basel. 2020;10(18). doi: ARTN 6296 10.3390/app10186296. PubMed PMID: WOS:000580451100001.
20. Citak-Er F, Firat Z, Kovanlikaya I, Ture U, Ozturk-Isik E. Machine-learning in grading of gliomas based on multi-parametric magnetic resonance imaging at 3T. Comput Biol Med. 2018;99:154-60. doi: 10.1016/j.compbiomed.2018.06.009. PubMed PMID: WOS:000442978700014.
21. Constantin A, Elkhaled A, Jalbert L, Srinivasan R, Cha S, Chang SM, et al. Identifying malignant transformations in recurrent low grade gliomas using high resolution magic angle spinning spectroscopy. Artificial Intelligence in Medicine. 2012;55(1):61-70. doi: 10.1016/j.artmed.2012.01.002. PubMed PMID: WOS:000303031000006.
22. Cui G, Jeong J, Lei Y, Wang T, Dong X, Liu T, et al. Machine-Learning-Based Classification of Low-Grade and High-Grade Glioblastoma Using Radiomic Features in Multiparametric MRI. Medical Physics. 2018;45(6):E617-E. PubMed PMID: WOS:000434978004360.
23. Cui G, Jeong JJ, Lei Y, Wang TH, Liu T, Curran WJ, et al. Machine-learning-based classification of Glioblastoma in multiparametric MRI. Proc Spie. 2019;10950. doi: Unsp 1095048 10.1117/12.2513110. PubMed PMID: WOS:000491309500143.
24. Dandil E, Bicer A. Automatic grading of brain tumours using LSTM neural networks on magnetic resonance spectroscopy signals. Iet Image Process. 2020;14(10):1967-79. doi: 10.1049/iet-ipr.2019.1416. PubMed PMID: WOS:000583360400004.
25. De Looze C, Beausang A, Cryan J, Loftus T, Buckley PG, Farrell M, et al. Machine learning: a useful radiological adjunct in determination of a newly diagnosed glioma's grade and IDH status. J Neuro-Oncol. 2018;139(2):491-9. doi: 10.1007/s11060-018-2895-4. PubMed PMID: WOS:000441544800028.
26. Decuyper M, Bonte S, Van Holen R. Binary Glioma Grading: Radiomics versus Pre-trained CNN Features. Medical Image Computing and Computer Assisted Intervention, Pt Iii. 2018;11072:498-505. doi: 10.1007/978-3-030-00931-1_57. PubMed PMID: WOS:000477769700057.
27. Devos A, Simonetti AW, van der Graaf M, Lukas L, Suykens JAK, Vanhamme L, et al. The use of multivariate MR imaging intensities versus metabolic data from MR spectroscopic imaging for brain tumour classification. J Magn Reson. 2005;173(2):218-28. doi: 10.1016/j.jmr.2004.12.007. PubMed PMID: WOS:000228076400005.
28. Dong F, Li Q, Xu D, Xiu WJ, Zeng Q, Zhu XL, et al. Differentiation between pilocytic astrocytoma and glioblastoma: a decision tree model using contrast-enhanced magnetic resonance imaging-derived quantitative radiomic features. European Radiology. 2019;29(8):3968-75. doi: 10.1007/s00330-018-5706-6. PubMed PMID: WOS:000473737100002.
29. Emblem KE, Zoellner FG, Tennoe B, Nedregaard B, Nome T, Due-Tonnessen P, et al. Predictive modeling in glioma grading from MR perfusion images using support vector machines. Magnetic Resonance in Medicine. 2008;60(4):945-52. doi: 10.1002/mrm.21736. PubMed PMID: WOS:000259651200022.
30. Gao M, Huang SY, Pan XQ, Liao X, Yang R, Liu J. Machine Learning-Based Radiomics Predicting Tumor Grades and Expression of Multiple Pathologic Biomarkers in Gliomas. Frontiers in Oncology. 2020;10. doi: ARTN 1676 10.3389/fonc.2020.01676. PubMed PMID: WOS:000575931600001.
31. Gates EDH, Lin JS, Weinberg JS, Prabhu SS, Hamilton J, Hazle JD, et al. Imaging-Based Algorithm for the Local Grading of Glioma. Am J Neuroradiol. 2020;41(3):400-7. doi: 10.3174/ajnr.A6405. PubMed PMID: WOS:000521965200012.
32. Ge C, Gu IY, Jakola AS, Yang J. Deep Learning and Multi-Sensor Fusion for Glioma Classification Using Multistream 2D Convolutional Networks. Annu Int Conf IEEE Eng Med Biol Soc. 2018;2018:5894-7. doi: 10.1109/EMBC.2018.8513556. PubMed PMID: 30441677.
33. Ge CJ, Gu IYH, Jakola AS, Yang J. Deep semi-supervised learning for brain tumor classification. Bmc Med Imaging. 2020;20(1). doi: ARTN 87 10.1186/s12880-020-00485-0. PubMed PMID: WOS:000557696800002.
34. Ge CJ, Qu QX, Gu IYH, Jakola AS. 3d Multi-Scale Convolutional Networks for Glioma Grading Using Mr Images. Ieee Image Proc. 2018:141-5. PubMed PMID: WOS:000455181500029.
35. Gutta S, Acharya J, Shiroishi MS, Hwang D, Nayak KS. Improved Glioma Grading Using Deep Convolutional Neural Networks. Am J Neuroradiol. 2021;42(2):233-9. doi: 10.3174/ajnr.A6882. PubMed PMID: WOS:000640504000009.
36. Haubold J, Demircioglu A, Gratz M, Glas M, Wrede K, Sure U, et al. Non-invasive tumor decoding and phenotyping of cerebral gliomas utilizing multiparametric F-18-FET PET-MRI and MR Fingerprinting. Eur J Nucl Med Mol I. 2020;47(6):1435-45. doi: 10.1007/s00259-019-04602-2. PubMed PMID: WOS:000529350000016.
37. Hedyehzadeh M, Nezhad SYD, Safdarian N. Evaluation of Conventional Machine Learning Methods for Brain Tumour Type Classification. Cr Acad Bulg Sci. 2020;73(6):856-65. doi: 10.7546/Crabs.2020.06.14. PubMed PMID: WOS:000546728400014.
38. Hsieh KLC, Tsai RJ, Teng YC, Lo CM. Effect of a computer-aided diagnosis system on radiologists' performance in grading gliomas with MRI. Plos One. 2017;12(2). doi: ARTN e0171342 10.1371/journal.pone.0171342. PubMed PMID: WOS:000396161700063.
39. Inano R, Oishi N, Kunieda T, Arakawa Y, Yamao Y, Shibata S, et al. Voxel-based clustered imaging by multiparameter diffusion tensor images for glioma grading. Neuroimage-Clin. 2014;5:396-407. doi: 10.1016/j.nicl.2014.08.001. PubMed PMID: WOS:000349667800043.
40. Jeong J, Wang LY, Ji B, Lei Y, Ali A, Liu T, et al. Machine-learning based classification of glioblastoma using delta-radiomic features derived from dynamic susceptibility contrast enhanced magnetic resonance images. Quant Imag Med Surg. 2019;9(7):1201-13. doi: 10.21037/qims.2019.07.01. PubMed PMID: WOS:000477984600002.
41. Ji B, Wang SL, Liu Z, Weinberg BD, Yang XF, Liu TM, et al. Revealing hemodynamic heterogeneity of gliomas based on signal profile features of dynamic susceptibility contrast-enhanced MRI. Neuroimage-Clin. 2019;23. doi: ARTN 101864 10.1016/j.nicl.2019.101864. PubMed PMID: WOS:000485804400075.
42. Kaur T, Saini BS, Gupta S. An optimal spectroscopic feature fusion strategy for MR brain tumor classification using Fisher Criteria and Parameter-Free BAT optimization algorithm. Biocybern Biomed Eng. 2018;38(2):409-24. doi: 10.1016/j.bbe.2018.02.008. PubMed PMID: WOS:000432621000016.
43. Khawaldeh S, Pervaiz U, Rafiq A, Alkhawaldeh RS. Noninvasive Grading of Glioma Tumor Using Magnetic Resonance Imaging with Convolutional Neural Networks. Appl Sci-Basel. 2018;8(1). doi: ARTN 27 10.3390/app8010027. PubMed PMID: WOS:000424388800027.
44. Koyuncu H, Barstugan M, Ozic MU. A comprehensive study of brain tumour discrimination using phase combinations, feature rankings, and hybridised classifiers. Med Biol Eng Comput. 2020;58(12):2971-87. doi: 10.1007/s11517-020-02273-y. PubMed PMID: WOS:000574740300001.
45. Kumar R, Gupta A, Arora HS, Pandian GN, Raman B. CGHF: A Computational Decision Support System for Glioma Classification Using Hybrid Radiomics- and Stationary Wavelet-Based Features. Ieee Access. 2020;8:79440-58. doi: 10.1109/Access.2020.2989193. PubMed PMID: WOS:000549839700017.
46. Lo CM, Chen YC, Weng RC, Hsieh KLC. Intelligent Glioma Grading Based on Deep Transfer Learning of MRI Radiomic Features. Appl Sci-Basel. 2019;9(22). doi: ARTN 4926 10.3390/app9224926. PubMed PMID: WOS:000502570800203.
47. Ma LF, Xiao Z, Li KL, Li SL, Li JL, Yi XP. Game theoretic interpretability for learning based preoperative gliomas grading. Future Gener Comp Sy. 2020;112:1-10. doi: 10.1016/j.future.2020.04.038. PubMed PMID: WOS:000567826800001.
48. Muneer KVA, Rajendran VR, Joseph KP. Glioma Tumor Grade Identification Using Artificial Intelligent Techniques. J Med Syst. 2019;43(5). doi: ARTN 113 10.1007/s10916-019-1228-2. PubMed PMID: WOS:000462197900002.
49. Mzoughi H, Njeh I, Wali A, Ben Slima M, BenHamida A, Mhiri C, et al. Deep Multi-Scale 3D Convolutional Neural Network (CNN) for MRI Gliomas Brain Tumor Classification. Journal of Digital Imaging. 2020;33(4):903-15. doi: 10.1007/s10278-020-00347-9. PubMed PMID: WOS:000534724900001.
50. Nakamoto T, Takahashi W, Haga A, Takahashi S, Kiryu S, Nawa K, et al. Prediction of malignant glioma grades using contrast-enhanced T1-weighted and T2-weighted magnetic resonance images based on a radiomic analysis. Sci Rep-Uk. 2019;9. doi: ARTN 19411 10.1038/s41598-019-55922-0. PubMed PMID: WOS:000508836900005.
51. Naser MA, Deen MJ. Brain tumor segmentation and grading of lower-grade glioma using deep learning in MRI images. Comput Biol Med. 2020;121. doi: ARTN 103758 10.1016/j.compbiomed.2020.103758. PubMed PMID: WOS:000542187300003.
52. Park YW, Choi YS, Ahn SS, Chang JH, Kim SH, Lee SK. Radiomics MRI Phenotyping with Machine Learning to Predict the Grade of Lower-Grade Gliomas: A Study Focused on Nonenhancing Tumors. Korean Journal of Radiology. 2019;20(9):1381-9. doi: 10.3348/kjr.2018.0814. PubMed PMID: WOS:000483930900006.
53. Polly FP, Shil SK, Hossain MA, Ayman A, Jang YM. Detection and Classification of HGG and LGG Brain Tumor Using Machine Learning. 2018 32nd International Conference on Information Networking (Icoin). 2018:813-7. PubMed PMID: WOS:000468812000158.
54. Qi C, Li YM, Fan X, Jiang Y, Wang R, Yang S, et al. A quantitative SVM approach potentially improves the accuracy of magnetic resonance spectroscopy in the preoperative evaluation of the grades of diffuse gliomas. Neuroimage-Clin. 2019;23. doi: ARTN 101835 10.1016/j.nicl.2019.101835. PubMed PMID: WOS:000485804400025.
55. Rajasree R, Columbus CC, Shilaja C. Multiscale-based multimodal image classification of brain tumor using deep learning method. Neural Comput Appl. 2021;33(11):5543-53. doi: 10.1007/s00521-020-05332-5. PubMed PMID: WOS:000570842500003.
56. Ranjith G, Parvathy R, Vikas V, Chandrasekharan K, Nair S. Machine learning methods for the classification of gliomas: Initial results using features extracted from MR spectroscopy. Neuroradiol J. 2015;28(2):106-11. doi: 10.1177/1971400915576637. PubMed PMID: WOS:000359429600003.
57. Reza SMS, Samad MD, Shboul ZA, Jones KA, Iftekharuddin KM. Glioma grading using structural magnetic resonance imaging and molecular data. J Med Imaging. 2019;6(2). doi: Artn 024501 10.1117/1.Jmi.6.2.024501. PubMed PMID: WOS:000481890500019.
58. Sajjad M, Khan S, Muhammad K, Wu WQ, Ullah A, Baik SW. Multi-grade brain tumor classification using deep CNN with extensive data augmentation. J Comput Sci-Neth. 2019;30:174-82. doi: 10.1016/j.jocs.2018.12.003. PubMed PMID: WOS:000457951200016.
59. Saxena N, Sharma R, Joshi K, Rana HS. Identification of Glioma from MR Images Using Convolutional Neural Network. Adv Intell Syst. 2019;880:589-97. doi: 10.1007/978-3-030-02686-8_44. PubMed PMID: WOS:000505677000044.
60. Sengupta A, Ramaniharan AK, Gupta RK, Agarwal S, Singh A. Glioma grading using a machine-learning framework based on optimized features obtained from T-1 perfusion MRI and volumes of tumor components. Journal of Magnetic Resonance Imaging. 2019;50(4):1295-306. doi: 10.1002/jmri.26704. PubMed PMID: WOS:000486307100029.
61. Sharif MI, Li JP, Khan MA, Saleem MA. Active deep neural network features selection for segmentation and recognition of brain tumors using MRI images. Pattern Recogn Lett. 2020;129:181-9. doi: 10.1016/j.patrec.2019.11.019. PubMed PMID: WOS:000504641500026.
62. Subashini MM, Sahoo SK, Sunil V, Easwaran S. A non-invasive methodology for the grade identification of astrocytoma using image processing and artificial intelligence techniques. Expert Syst Appl. 2016;43:186-96. doi: 10.1016/j.eswa.2015.08.036. PubMed PMID: WOS:000365058700018.
63. Sudre CH, Panovska-Griffiths J, Sanverdi E, Brandner S, Katsaros VK, Stranjalis G, et al. Machine learning assisted DSC-MRI radiomics as a tool for glioma classification by grade and mutation status. Bmc Med Inform Decis. 2020;20(1). doi: ARTN 149 10.1186/s12911-020-01163-5. PubMed PMID: WOS:000549153000001.
64. Sun P, Wang DF, Mok VCT, Shi L. Comparison of Feature Selection Methods and Machine Learning Classifiers for Radiomics Analysis in Glioma Grading. Ieee Access. 2019;7:102010-20. doi: 10.1109/Access.2019.2928975. PubMed PMID: WOS:000481688500124.
65. Takahashi S, Takahashi W, Tanaka S, Haga A, Nakamoto T, Suzuki Y, et al. Radiomics Analysis for Glioma Malignancy Evaluation Using Diffusion Kurtosis and Tensor Imaging. Int J Radiat Oncol. 2019;105(4):784-91. doi: 10.1016/j.ijrobp.2019.07.011. PubMed PMID: WOS:000491350500017.
66. Tian Q, Yan LF, Zhang X, Zhang X, Hu YC, Han Y, et al. Radiomics strategy for glioma grading using texture features from multiparametric MRI. Journal of Magnetic Resonance Imaging. 2018;48(6):1518-28. doi: 10.1002/jmri.26010. PubMed PMID: WOS:000451113500007.
67. Tian ZR, Chen CY, Fan YM, Ou XJ, Wang J, Ma XL, et al. Glioblastoma and Anaplastic Astrocytoma: Differentiation Using MRI Texture Analysis. Frontiers in Oncology. 2019;9. doi: ARTN 876 10.3389/fonc.2019.00876. PubMed PMID: WOS:000484510900001.
68. Ural B, Ozisik P, Hardalac F. An improved computer based diagnosis system for early detection of abnormal lesions in the brain tissues with using magnetic resonance and computerized tomography images. Multimed Tools Appl. 2020;79(21-22):15613-34. doi: 10.1007/s11042-019-07823-7. PubMed PMID: WOS:000538675900068.
69. Vamvakas A, Williams SC, Theodorou K, Kapsalaki E, Fountas K, Kappas C, et al. Imaging biomarker analysis of advanced multiparametric MRI for glioma grading. Phys Medica. 2019;60:188-98. doi: 10.1016/j.ejmp.2019.03.014. PubMed PMID: WOS:000464560200026.
70. Van Cauter S, De Keyzer F, Sima DM, Sava AC, D'Arco F, Veraart J, et al. Integrating diffusion kurtosis imaging, dynamic susceptibility-weighted contrast-enhanced MRI, and short echo time chemical shift imaging for grading gliomas. Neuro-Oncology. 2014;16(7):1010-21. doi: 10.1093/neuonc/not304. PubMed PMID: WOS:000338126100014.
71. Wiestler B, Kluge A, Lukas M, Gempt J, Ringel F, Schlegel J, et al. Multiparametric MRI-based differentiation of WHO grade II/III glioma and WHO grade IV glioblastoma. Sci Rep-Uk. 2016;6. doi: ARTN 35142 10.1038/srep35142. PubMed PMID: WOS:000385298400001.
72. Wu JF, Qian ZY, Tao L, Yin JH, Ding SW, Zhang YM, et al. Resting state fMRI feature-based cerebral glioma grading by support vector machine. Int J Comput Ass Rad. 2015;10(7):1167-74. doi: 10.1007/s11548-014-1111-z. PubMed PMID: WOS:000357278000015.
73. Wu YP, Hao HH, Li J, Wu WG, Lin YS, Wang MY. Four-Sequence Maximum Entropy Discrimination Algorithm for Glioma Grading. Ieee Access. 2019;7:52246-56. doi: 10.1109/Access.2019.2910849. PubMed PMID: WOS:000466586000001.
74. Wu YP, Liu B, Wu WG, Lin YS, Yang C, Wang MY. Grading glioma by radiomics with feature selection based on mutual information. J Amb Intel Hum Comp. 2018;9(5):1671-82. doi: 10.1007/s12652-018-0883-3. PubMed PMID: WOS:000444823600028.
75. Yang Y, Yan LF, Zhang X, Han Y, Nan HY, Hu YC, et al. Glioma Grading on Conventional MR Images: A Deep Learning Study With Transfer Learning. Front Neurosci-Switz. 2018;12. doi: ARTN 804 10.3389/fnins.2018.00804. PubMed PMID: WOS:000450198700001.
76. Yang Y, Yan LF, Zhang X, Nan HY, Hu YC, Han Y, et al. Optimizing Texture Retrieving Model for Multimodal MR Image-Based Support Vector Machine for Classifying Glioma. Journal of Magnetic Resonance Imaging. 2019;49(5):1263-74. doi: 10.1002/jmri.26524. PubMed PMID: WOS:000464390200006.
77. Ye CZ, Yang J, Geng DY, Zhou Y, Chen NY. Fuzzy rules to predict degree of malignancy in brain glioma. Med Biol Eng Comput. 2002;40(2):145-52. doi: Doi 10.1007/Bf02348118. PubMed PMID: WOS:000175904800001.
78. Zhan TM, Feng PP, Hong XN, Lu ZY, Xiao L, Zhang YD. An automatic glioma grading method based on multi-feature extraction and fusion. Technol Health Care. 2017;25:S377-S85. doi: 10.3233/Thc-171341. PubMed PMID: WOS:000406157200042.
79. Zhang X, Yan LF, Hu YC, Li G, Yang Y, Han Y, et al. Optimizing a machine learning based glioma grading system using multi-parametric MRI histogram and texture features. Oncotarget. 2017;8(29):47816-30. doi: 10.18632/oncotarget.18001. PubMed PMID: WOS:000405694000091.
80. Zhang Y, Chen CY, Cheng YF, Teng YE, Guo W, Xu H, et al. Ability of Radiomics in Differentiation of Anaplastic Oligodendroglioma From Atypical Low-Grade Oligodendroglioma Using Machine-Learning Approach. Frontiers in Oncology. 2019;9. doi: ARTN 1371 10.3389/fonc.2019.01371. PubMed PMID: WOS:000519586000001.
81. Zhang ZW, Xiao JJ, Wu SD, Lv FJ, Gong JW, Jiang L, et al. Deep Convolutional Radiomic Features on Diffusion Tensor Images for Classification of Glioma Grades. Journal of Digital Imaging. 2020;33(4):826-37. doi: 10.1007/s10278-020-00322-4. PubMed PMID: WOS:000516066800002.
82. Zhao SS, Feng XL, Hu YC, Han Y, Tian Q, Sun YZ, et al. Better efficacy in differentiating WHO grade II from III oligodendrogliomas with machine-learning than radiologist's reading from conventional T1 contrast-enhanced and fluid attenuated inversion recovery images. Bmc Neurol. 2020;20(1). doi: ARTN 48 10.1186/s12883-020-1613-y. PubMed PMID: WOS:000513154000001.
83. Zhuge Y, Ning H, Mathen P, Cheng JY, Krauze AV, Camphausen K, et al. Automated glioma grading on conventional MRI images using deep convolutional neural networks. Medical Physics. 2020;47(7):3044-53. doi: 10.1002/mp.14168. PubMed PMID: WOS:000531361600001.
84. Zollner FG, Emblem KE, Schad LR. Support Vector Machines in DSC-Based Glioma Imaging: Suggestions for Optimal Characterization. Magnetic Resonance in Medicine. 2010;64(4):1230-6. doi: 10.1002/mrm.22495. PubMed PMID: WOS:000282477100034.
85. Zollner FG, Emblem KE, Schad LR. SVM-based glioma grading: Optimization by feature reduction analysis. Zeitschrift Fur Medizinische Physik. 2012;22(3):205-14. doi: 10.1016/j.zemedi.2012.03.007. PubMed PMID: WOS:000310419800005.
86. Kumar V, Gu Y, Basu S, Berglund A, Eschrich SA, Schabath MB, et al. Radiomics: the process and the challenges. Magn Reson Imaging. 2012;30(9):1234-48. doi: 10.1016/j.mri.2012.06.010. PubMed PMID: 22898692; PubMed Central PMCID: PMC3563280.

**Appendix A5. TRIPOD.**

**Table A8. Individual TRIPOD Item Explanations and Adherence Rates.**

| **TRIPOD Item** | | **Description** | **% of Studies Adhering (*n*)** |
| --- | --- | --- | --- |
| *Title and Abstract* | | | |
| Title | 1 | Identify the study as developing a multivariable prediction model, the target population, and the outcome to be predicted. | 0% (*0*) |
| Abstract | 2 | Provide a summary of objectives, study design, setting, participants, sample size, predictors, outcome, statistical analysis, results, and conclusions. | 0% (*0*) |
| *Introduction* | | | |
| Background and objectives | 3a | Explain the medical context and rationale for developing the multivariable prediction model, including references to existing models. | 98.8% (*84*) |
|  | 3b | Specify the objectives, including whether the study describes the development or validation of the model or both. | 95.3% (*81*) |
| *Methods* | | | |
| Source of data | 4a | Describe the study design or source of data (e.g., randomize trial, cohort, or registry data). | 96.5% (*82*) |
|  | 4b | Specify the key study dates, including start of accrual; end of accrual; and, if applicable, end of follow-up. | 21.2% (*18*) |
| Participants | 5a | Specify key elements of the study setting (e.g., primary care, secondary care, general population) including number and location of centers. | 48.2% (*41*) |
|  | 5b | Describe eligibility criteria for participants. | 45.9% (*39*) |
| Outcome | 6a | Clearly define the outcome that is predicted by the prediction model, including how and when assessed. | 22.4% (*19*) |
|  | 6b | Report any actions to blind assessment of the outcome to be predicted. | 8.2% (*7*) |
| Predictors | 7a | Clearly define all predictors used in developing the multivariable prediction model, including how and when they were measured. | 54.1% (*46*) |
|  | 7b | Report any actions to blind assessment of predictors for the outcome and other predictors. | 71.8% (*61*) |
| Sample size | 8 | Explain how the study size was arrived at. | 2.4% (*2*) |
| Missing data | 9 | Describe how missing data were handled (e.g., complete-case analysis, single imputation, multiple imputation) with details of any imputation method. | 12.9% (*11*) |
| Statistical analysis methods | 10a | Describe how predictors were handled in the analyses. | 85.9% (*73*) |
|  | 10b | Specify type of model, all model-building procedures (including any predictor selection), and method for internal validation. | 74.1% (*63*) |
|  | 10d | Specify all measures used to assess model performance. | 8.3% (*7*) |
| *Results* | | | |
| Participants | 13a | Describe the flow of participants through the study, including the number of participants with and without the outcome. | 41.2% (*35*) |
|  | 13b | Describe the characteristics of the participants (basic demographics, clinical features, available predictors), including the number of participants with missing data for predictors and outcome. | 7.1% (*6*) |
| Model development | 14a | Specify the number of participants and outcome events in each analysis. | 94.1% (*80*) |
| Model specification | 15a | Present the full prediction model to allow predictions for individuals (i.e., all regression coefficients, and model intercept or baseline survival at a given time point). | 2.4% (*2*) |
|  | 15b | Explain how to use the prediction model. | 41.2% (*35*) |
| Model performance | 16 | Report performance measures (with confidence intervals) for the prediction model. | 0% (*0*) |
| *Discussion* | | | |
| Limitations | 18 | Discuss any limitations of the study (such as nonrepresentative sample, few events per predictor, missing data). | 77.6% (*66*) |
| Interpretation | 19b | Give an overall interpretation of the results considering objectives, limitations, results from similar studies and other relevant evidence. | 100% (*85*) |
| Implications | 20 | Discuss the potential clinical use of the model and implications for future research. | 82.4% (*70*) |
| Other information | 22 | Give the source of funding and the role of the funders for the present study. | 9.5% (*8*) |
